# Colon adenocarcinoma-derived cells that express induced-pluripotent stem cell markers possess stem cell function

**DOI:** 10.1371/journal.pone.0232934

**Published:** 2020-05-19

**Authors:** Matthew J. Munro, Lifeng Peng, Susrutha K. Wickremesekera, Swee T. Tan

**Affiliations:** 1 Gillies McIndoe Research Institute, Wellington, New Zealand; 2 School of Biological Sciences and Centre for Biodiscovery, Victoria University of Wellington, Wellington, New Zealand; 3 Department of General Surgery, Upper Gastrointestinal, Hepatobiliary & Pancreatic Section, Wellington Regional Hospital, Wellington, New Zealand; 4 Wellington Regional Plastic, Maxillofacial & Burns Unit, Hutt Hospital, Lower Hutt, New Zealand; 5 Department of Surgery, The University of Melbourne, Melbourne, Victoria, Australia; National Institute of technology Rourkela, INDIA

## Abstract

**Aims:**

Much work has been done to find markers of cancer stem cells (CSCs) that distinguish them from the tumor bulk cells and normal cells. Recent CSC research has applied the induced pluripotent stem cell (iPSC) concept. In this study, we investigated the expression of a panel of iPSC markers in primary colon adenocarcinoma (CA)-derived cell lines.

**Materials and methods:**

Expression of iPSC markers by CA-derived primary cell lines was interrogated using immunocytochemistry, western blotting and RT-qPCR. The stem cell function of these cells was then assessed *in vitro* using differentiation and tumorsphere assays.

**Results:**

Expression of iPSC markers OCT4, SOX2, NANOG, KLF4 and c-MYC was more widespread in high-grade CA (HGCA) cell lines than low-grade CA (LGCA) cell lines, as demonstrated by western blotting and RT-qPCR. These cells could be induced to differentiate down the three embryonic lineages. Cells derived from HGCA were more capable of forming tumorspheres than those derived from LGCA. EpCAM sorting revealed that a population enriched for EpCAM^High^ cells formed larger tumorspheres than EpCAM^Low^ cells. Pluripotency markers, SSEA4 and TRA-1-60, were co-expressed by a small subpopulation of cells that also co-expressed SOX2 in 75% and OCT4 in 50% of the cell lines.

**Conclusions:**

CA-derived primary cell lines contain tumorsphere-forming cells which express key pluripotency genes and can differentiate down 3 embryonic lineages, suggesting a pluripotent CSC-like phenotype. There appear to be two iPSC-like subpopulations, one with high EpCAM expression which forms larger tumorspheres than another with low EpCAM expression. Furthermore, these cells can be characterized based on iPSC marker expression, as we have previously demonstrated in the original CA tumor tissues.

## Introduction

The cancer stem cell (CSC) concept proposes that tumor growth, metastasis and recurrence are driven by CSCs, a subpopulation of cancer cells capable of dividing asymmetrically to produce identical CSCs as well as differentiated cancer cells [1–4]. Identification and isolation of CSCs have proven challenging due to their heterogeneity and similarity to normal somatic stem cells. There has been recent focus on finding markers that identify and characterize CSCs and serve as therapeutic targets.

As CSCs are pluripotent, research into them has begun to utilize induced pluripotent stem cell (iPSC) research. The first iPSCs were successfully produced by Yamanaka and Takahashi in 2006 by introducing the *OCT4*, *SOX2*, *KLF4* and *c-MYC* genes into mature mouse fibroblasts [5], and adult human fibroblasts in 2007 [6, 7]. In the same year, the Thomson laboratory achieved a similar outcome by using *NANOG* and *LIN28* in the place of *KLF4* and *c-MYC* [8].

OCT4, SOX2 and NANOG are responsible for maintaining pluripotency [9, 10]. SOX2 regulates OCT4 expression, and together with OCT4 forms a complex which allows transcription of *NANOG* [11, 12]. KLF4 is a marker of differentiation down the goblet cell lineage of intestinal epithelial cells, and is also associated with sphere formation, pluripotency and self-renewal in colon cancer cells [13–15]. c-MYC is a proto-oncogene which is implicated in many diseases, including colon cancer in which it confers a poor prognosis and heightened progression [7].

It has been hypothesized that these drivers of pluripotency in embryonic stem cells (ESCs) may be used to identify subpopulations of CSCs. Based on the protein expression patterns of iPSC markers OCT4, SOX2, NANOG, KLF4 and c-MYC, we have demonstrated the presence of two unique subpopulations of CSCs in primary colon adenocarcinoma (CA): an epithelial subpopulation expressing NANOG, SOX2 and KLF4, and a stromal subpopulation expressing OCT4, SOX2 and c-MYC [16]. Furthermore, we have performed immunohistochemical (IHC) staining to ensure that in all cancer cases, EpCAM expression is restricted to epithelial cells. In this study, we were interested to see whether we could isolate these distinct subpopulations for further analysis.

There are now a range of validated *in vitro* tests for pluripotency which mitigate reliance on animal testing, with its inherent ethical considerations, and teratoma assays, which are not standardized and therefore inconsistent. In place of *in vivo* work, stem cell function is typically demonstrated by confirmation of pluripotency marker expression, tumorsphere formation assays and multilineage differentiation capability. Here, we used primary cell lines derived from CA tissue samples included in our recent study [16] to assess their expression of the iPSC markers and test their stem cell functionality.

## Materials and methods

### Cell culture

Primary cell lines derived from 3 low-grade (LG) and 3 high-grade (HG) CA tissue samples included in our previous study [16] were provided by the Gillies McIndoe Research Institute Tissue Bank for this study with approval by the Central Health and Disability Ethics Committee (Ref. 15/CEN/106). Written consent was obtained from all participants. Commercial cell lines were used as positive controls for tumorsphere formation (CaCo2; cat# HTB-37, ATCC, In Vitro Technologies, Auckland, New Zealand), differentiation and α-SMA expression (3T3; cat# CRL-1658, ATCC), EpCAM expression (HepG2; cat# HB8025, ATCC) and iPSC marker expression (NTERA-2; cat# CRL-1973, ATCC).

Cells were cultured in Nunc™ EasYFlasks™ (Thermo Fisher Scientific, Waltham, MA, USA) using DMEM media with high glucose concentration and containing pyruvate (cat# 10569010, Thermo), and supplemented with 10% fetal calf serum (FCS; cat# 10091148, Thermo), 5% mTeSR Complete (cat# 85850, STEMCELL Technologies, Tullamarine, VIC, Australia), 1% penicillin/streptomycin (cat# 15140122, Thermo) and 0.2% gentamicin/amphotericin B (cat# R01510 Thermo).

Cells were passaged upon reaching 75–95% confluency using PBS (cat# 70013032, Thermo) to wash the cells and TrypLE Express Enzyme (cat# 12605093, Thermo) to detach them from the flask.

### Cell sorting

The CA-derived primary cell lines were sorted into EpCAM^High^ and EpCAM^Low^ subpopulations using the CELLection™ Epithelial Enrich Dynabeads kit (cat# 16203, Thermo). Cells were lifted from their culture flask using TrypLE and a cell count was performed to ensure there were between 1x10^6^ and 2x10^7^ live cells. Cells were centrifuged at 500 x g for 5 min and the cell pellet was resuspended in 1 mL of PBS with 0.1% FCS in a 15 mL Falcon tube. Fifty μL of washed Dynabeads were added, and the tube was incubated for 30 min at 4°C with gentle tilting. Following incubation, the tube was placed in a DynaMag magnet (Thermo) for 2 min, and the supernatant containing unbound EpCAM^Low^ cells was transferred to a new tube. The incubation tube was then removed from the magnet and the beads were washed gently with 1 mL of PBS with 0.1% FCS before being returned to the magnet for 2 min. This supernatant was pooled with the former supernatant, and a total of 3 washes were performed in this manner. After 3 washes, the beads were resuspended in 200 μL of DMEM with 1% FCS and 4 μL of Release Enzyme Buffer and incubated at room temperature for 15 min with gentle tilting and rotation to release the bound cells. The tube was placed in the DynaMag for 2 min and the supernatant containing unbound EpCAM^High^ cells was transferred to a new tube. Wash steps as above were performed but using 200 μL of DMEM with 1% FCS, and supernatants were pooled for a cell count. These cells were then plated into an appropriately sized culture flask.

Cell counting was also performed on the collected supernatants containing EpCAM^Low^ cells, and these cells were plated to an appropriately sized culture flask.

### Immunocytochemistry

Immunocytochemistry (ICC) was performed using the pluripotent stem cell (PSC) 4-marker ICC Kit (cat# A24881, Thermo), which is an established method for identifying pluripotent cells. The marker proteins examined included two of the iPSC markers, OCT4 and SOX2, plus additional TRA-1-60 and SSEA-4. Cells were seeded onto 8-chamber culture slides (cat# 354118, Corning, In Vitro Technologies) at a density of 5000 cells per well. After allowing cells to reach 75–95% confluency, growth medium was removed, and each well was washed once with PBS. Subsequently, 150 μL of Fixative Solution (cat# A24344, Thermo) was added for 15 min, before being removed and replaced with 150 μL of Permeabilization Solution S (cat# A24878, Thermo) for 15 min. Finally, 300 μL of Blocking Solution was added (cat# A24353, Thermo) for 30 min. These steps were carried out at room temperature.

Primary antibodies included rabbit anti-OCT4 (cat# A24867, Thermo), rat anti-SOX2 (cat# A24759, Thermo), mouse IgG3 anti-SSEA4 (cat# A24866, Thermo) and mouse IgM anti-TRA-1-60 (cat# A24868, Thermo). Primary antibodies were diluted 1:150 in Blocking Solution and the cells were incubated with these at 4°C overnight. After exposure to the primary antibodies, the cells were washed with 300 μL of Wash Buffer (cat# A24348, Thermo) 3 times for 2–3 min. Secondary antibodies included Alexa Fluor^®^ 555 donkey anti-rabbit (cat# A24869, Thermo) and goat anti-mouse IgM (cat# A24871, Thermo), Alexa Fluor^®^ 594 donkey anti-rabbit (cat # A24870, Thermo) and goat anti-mouse IgM (cat# A24872, Thermo), and Alexa Fluor^®^ 488 goat anti-mouse IgG3 (cat# A24877, Thermo) and donkey anti-rat (cat# A24872, Thermo).

**Fig 1:**
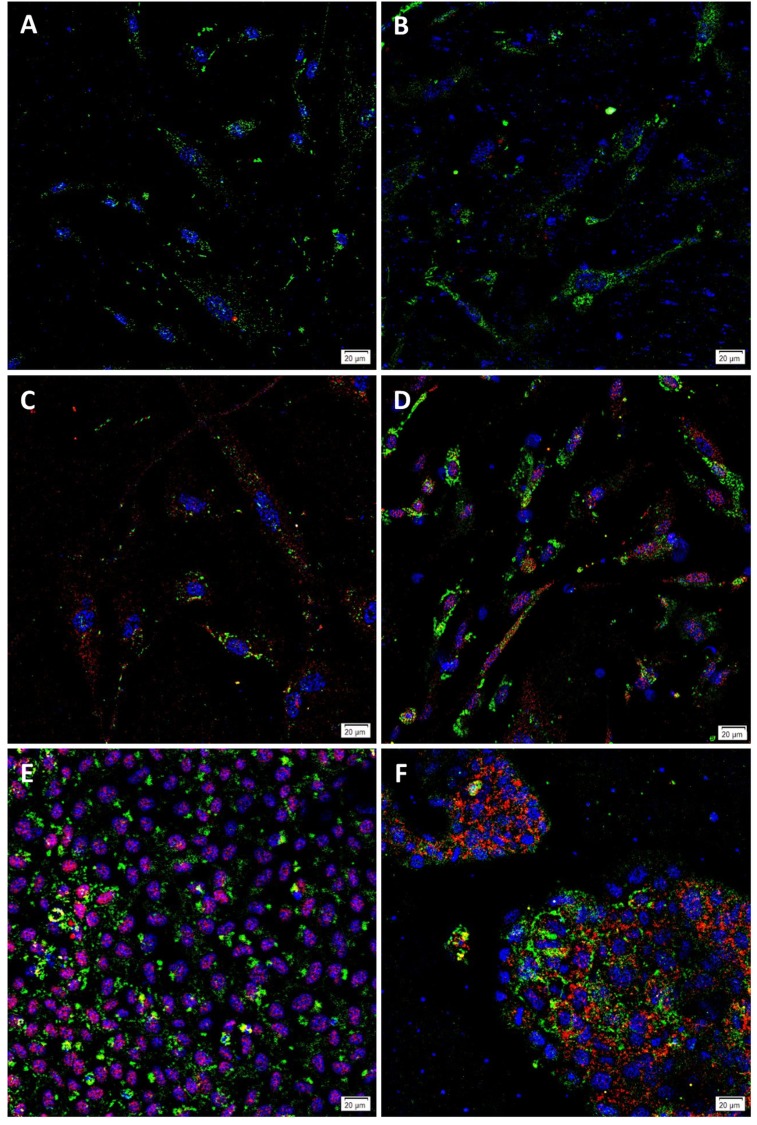
Representative immunofluorescence immunocytochemical images. EpCAM^Low^ (A) and EpCAM^High^ (B) cells from low-grade colon adenocarcinoma (LGCA)-derived primary cell lines, and EpCAM^Low^ (C) and EpCAM^High^ (D) cells from high-grade colon adenocarcinoma (HGCA)-derived primary cell lines showing expression of SSEA4 (green) and OCT4 (red). LGCA (n = 3); HGCA (n = 3). Positive control NTERA-2 (E) and CaCo2 (F) cells were stained for SSEA4 (green) and OCT4 (red). Original magnification: 400x; scale bar = 20 μm.

The required combinations of secondary antibodies were diluted 1:250 in Blocking Solution in which the cells were incubated for 1 h in the dark at room temperature. Following exposure to secondary antibodies, the cells were washed 3 times with Wash Buffer as above. Two drops per milliliter of NucBlue™ Fixed Cell nuclear stain (cat# R37606, Thermo) was added to the third wash and left on the cells for 5 min. Finally, the chambers were removed, and a coverslip was mounted using Histomount (cat# 008030, Thermo). Fluorescence was visualized using the FV1200 Laser Scanning Microscope (Olympus, Tokyo, Japan). NTERA-2 and CaCo2 cell lines were used as positive controls (Fig 1E and 1F). As a negative control, the primary antibodies were omitted, and cells were exposed to only the secondary antibodies (S1 Fig).

### Tumorsphere formation assays

Cells were lifted from their culture flask using TrypLE and a cell count was performed. Tumorsphere assays were carried out in Corning Costar 6-well ultra-low attachment plates (cat# 3471, In Vitro Technologies) or T25 Nunclon Sphera EasyFlasks (cat# 174951, Thermo). Cell pellets were resuspended in StemXVivo Serum-free Tumorsphere media (cat# CCM012, R&D Systems, In Vitro Technologies) and seeded at a density of 1x10^4^ live cells per milliliter, with 4 mL per well in a 6-well plate or 10 mL per T25 flask. Progress was checked daily using an inverted microscope, and fresh media was added every 3–4 d. Tumorspheres were measured using an inverted microscope and CellSens 2.0 software (Olympus). If tumorspheres had not formed by 14 d, the cells were harvested or discarded. A positive result for tumorsphere formation was assigned if the average diameter of measured spheres was at least 50 μm.

For passaging or harvesting, the tumorsphere media was aspirated and deposited into a 50 mL Falcon tube. Each plate or flask was rinsed with PBS and this wash solution was collected into the same tube to maximize yield. More PBS was added to the tube to achieve a 1:3 dilution of the viscous tumorsphere media in PBS. The diluted media was then passed through filters with 20 μm pores to collect tumorspheres and allow debris to pass through for disposal. Filters were rinsed thoroughly with PBS to retrieve the tumorspheres. The tubes containing tumorspheres were centrifuged and supernatant carefully removed, leaving 1–2 mL of supernatant. If harvesting for cryopreservation, the pellets were resuspended in 1 mL of PBS and transferred to a 1.5 mL microcentrifuge tube for pelleting and cryopreservation at -80°C. For sub-culturing, the pellet was resuspended in the remaining supernatant and 1mL of TrypLE was added and incubated for 2–3 min, pipetting up and down every 30 sec to ensure the cells remained in suspension. Any remaining aggregates were broken down by pipetting up and down 10–20 times. Immediately following this, 5 mL of PBS was added to dilute the TrypLE enzyme. Tubes were centrifuged and the supernatant was carefully removed, leaving 1–2 mL of supernatant. A live cell count was performed, and the cells were seeded in tumorsphere media at a density of 1x10^4^ live cells per milliliter in an appropriately sized low-attachment plate or flask. CaCo2 cells were used as a positive control for tumorsphere formation [17] (S4 Fig).

### Mesoderm differentiation assays

Cells were induced to undergo osteogenic differentiation using the StemPro^®^ Osteogenesis Differentiation Kit (cat# A1007201, Thermo). Cells were seeded onto 8-chamber culture slides (cat# 354118, Corning, In Vitro Technologies) at a density of 5,000 cells per well. After allowing 1–2 d to adhere, regular DMEM media was replaced with Osteogenesis Differentiation Medium which was replaced every 2–3 d for a total of 10–14 d. After the differentiation period, the media was removed, and the cells were fixed in 5% formalin for 5 min and then thoroughly washed with distilled water. Fixed cells were then stained with Alizarin Red solution (pH 4.2) for 5 min. Once the dye had been removed, cells were washed 5 times with distilled water and visualized under an inverted microscope. 3T3 and CaCo2 cells were used as positive controls for mesodermal differentiation (S9 Fig). As a negative control, cells grown in regular DMEM media were also stained with Alizarin Red (S10 Fig).

### Endoderm differentiation assays

Endoderm differentiation assays were performed using the StemXVivo^®^ Endoderm Kit (cat# SC019B, R&D Systems, In Vitro Technologies). Cells were seeded onto 8-chamber culture slides (cat# 354118, Corning, In Vitro Technologies) at a density of 5000 cells per well. After allowing 1–2 d to adhere, regular DMEM media was refreshed, with the addition of *b*FGF, and left for 4 h. Following this, the cells were washed with PBS and cultured in Differentiation Media I overnight. This was then replaced with Differentiation Media II, which was refreshed twice daily. After 2 d in Media II, the cells were washed with PBS and fixed using 10% formalin for 20 min at room temperature. Cells were washed 3 times using 1% BSA in PBS, and permeabilized using 5% BSA in PBS with 0.1% Tween-20 for 45 min at room temperature. Following this, anti-human SOX17 primary antibody was added to the permeabilization buffer (final concentration 10 μg/mL) and left in the fridge overnight. Cells were then washed 3 times using 1% BSA in PBS before being exposed to the NorthernLights™ fluorescent secondary antibody (cat# NL001, R&D Systems, In Vitro Technologies), at a 1:200 dilution in permeabilization buffer, for 1 h in the dark at room temperature. Cells were washed 3 times using 1% BSA in PBS. Two drops per milliliter of NucBlue™ nuclear stain was added to the third wash and left on the cells for 5 min. Finally, the chambers were removed, and a coverslip was mounted using Histomount (cat# 008030, Thermo). Fluorescence was visualized using the FV1200 Laser Scanning Microscope (Olympus). As a negative control, the primary antibodies were omitted, and cells were exposed to only the secondary antibodies (S11 Fig). Cells were also stained after growing in regular DMEM media to assess their intrinsic expression of SOX17 (S12 Fig). CaCo2 cells were also interrogated for their endodermal differentiation capacity (S13 Fig).

### Ectoderm differentiation assay

Ectoderm differentiation assays were performed using the StemXVivo^®^ Ectoderm Kit (cat# SC031B, R&D Systems, In Vitro Technologies). Cells were seeded onto 8-chamber culture slides at a density of 5000 cells per well. After allowing 1–2 d to adhere, regular DMEM media was replaced with Ectoderm Differentiation Media. This was refreshed once daily. After a total of 3 d in differentiation media, the cells were washed with PBS and fixed using 10% formalin for 20 min at room temperature. Cells were washed 3 times using 1% BSA in PBS and permeabilized using 5% BSA in PBS with 0.1% Tween-20, for 45 min at room temperature. Following this, anti-human Otx2 primary antibody was added to the permeabilization buffer (final concentration 10 μg/mL) and left at 4°C overnight. Cells were then washed 3 times using 1% BSA in PBS before being exposed to the NorthernLights™ fluorescent secondary antibody at a 1:200 dilution in permeabilization buffer for 1 h in the dark at room temperature. Cells were washed 3 times using 1% BSA in PBS. NucBlue™ nuclear stain was added to the third wash and left on the cells for 5 min. Finally, the chambers were removed, and a coverslip was mounted using Histomount. Fluorescence was visualized using the FV1200 Laser Scanning Microscope (Olympus). As a negative control, the primary antibodies were omitted, and cells were exposed to only the secondary antibodies (S14 Fig). Cells were also stained after growing in regular DMEM media to assess their intrinsic expression of Otx2 (S15 Fig). CaCo2 cells were also interrogated for their ectodermal differentiation capacity (S16 Fig).

### RT-qPCR

RNA extraction was performed using a QIAcube (Qiagen, VIC, Australia) and quantified using a NanoDrop 2000 (Thermo). RT-qPCR was carried out on a Rotor Gene Q (Qiagen) using the Qiagen RT-PCR Multiplex Kit (cat#204972, Qiagen). Taqman primers (Thermo Fisher) were used for OCT4 (Hs00999632_g1; 77kb), SOX2 (Hs01053049_s1; 91kb), NANOG (Hs04399610_g1; 101kb), KLF4 (Hs00358836_m1; 110kb), c-MYC (Hs00153408_m1; 107kb) and housekeeper *GAPDH* (Hs99999905_m1). Briefly, each PCR run included 15 min at 50°C and 5 min at 95°C, followed by 38 cycles of 95°C for 15 sec and either 60°C (NANOG, SOX2) or 62°C (OCT4, KLF4, c-MYC) for 15 sec. Each assay tube contained 20 ng of RNA. Due to the difficulties in culturing patient-matched normal colon-derived cell lines, RNA abundance for each gene of interest was measured relative to a commercial qPCR Human Reference Total RNA (cat# CLT636690, Mediray, Albany, Auckland, NZ).

### Western blotting

Total protein was extracted from the cells using RIPA buffer (cat# 89901, Thermo) and quantified by a BCA Protein Assay (cat# 23227, Thermo). Protein electrophoresis was performed on Bolt™ 4–12% Bis-Tris Plus gels (cat# NW04125BOX, Thermo) with 20 μg of proteins per lane and 2 μL of Kaleidoscope^™^ molecular weight marker (cat# 161–0375, Biorad, Rosedale, Auckland, NZ) in lane 1, for 50 min at 150V, 3A and 300W. The electrophoresed proteins were transferred to a PVDF membrane in an iBlot2 (Thermo) before exposure to primary and secondary antibodies in an iBind Flex (Thermo). Images were captured using a ChemiDoc MP Imaging System (Biorad) and ImageLab 6.0 software (Biorad).

Primary antibodies against OCT4 (1:500; cat# ab109183, Abcam, Melbourne, VIC, Australia), SOX2 (1:1000; cat#48–1400, Thermo), NANOG (1:1000; cat# ab109250, Abcam), KLF4 (1:1000; cat# NBP2-24749, Novus, In Vitro Technologies), c-MYC (1:1000; cat# ab32072, Abcam), EpCAM (1:1000; cat# ab71916, Abcam), α-SMA (1:2000; cat# ab5694, Abcam) and the housekeeping protein α-tubulin (1:2000; cat# ab7291, Abcam) were used. Secondary antibodies used were the chemiluminescent HRP-linked goat anti-rabbit (1:1000; cat# ab6721, Abcam) and fluorescent AlexaFluor488 donkey anti-mouse (1:1000; cat# A-21202, Thermo). Protein extracted from NTERA-2, HepG2 and 3T3 cells was used as positive controls.

Densitometry was performed on the images using Image Lab 6.0 (Biorad) for a semi-quantitative measure of protein abundance. Intensity values were normalized against the loading control α-tubulin. The mean intensity across the three biological replicates for each marker was calculated and graphed with error bars showing the standard deviation.

### Statistical analysis

Statistical analysis was performed on tumorsphere formation assay, RT-qPCR and western blotting (WB) data using GraphPad Prism version 8. Prism was used to calculate the average and standard deviation for maximum tumorsphere diameters. For RT-qPCR, variation between technical replicates within each biological replicate was assessed by calculating the mean, with 95% confidence intervals displayed as error bars. For densitometry data, two-tailed unpaired t-tests were performed to assess whether the expression levels of each marker between LG and HG or between EpCAM^low^ and EpCAM^high^ cells were significantly different (p<0.05).

## Results

### Immunocytochemistry

ICC was performed to assess the expression of pluripotency markers by CA-derived primary cell lines using the PSC 4-marker ICC kit, which includes two of our iPSC markers of interest, OCT4 and SOX2, as well as two validated pluripotency markers, SSEA4 and TRA-1-60, to verify if cells expressing OCT4 and SOX2 were pluripotent.

**Fig 2:**
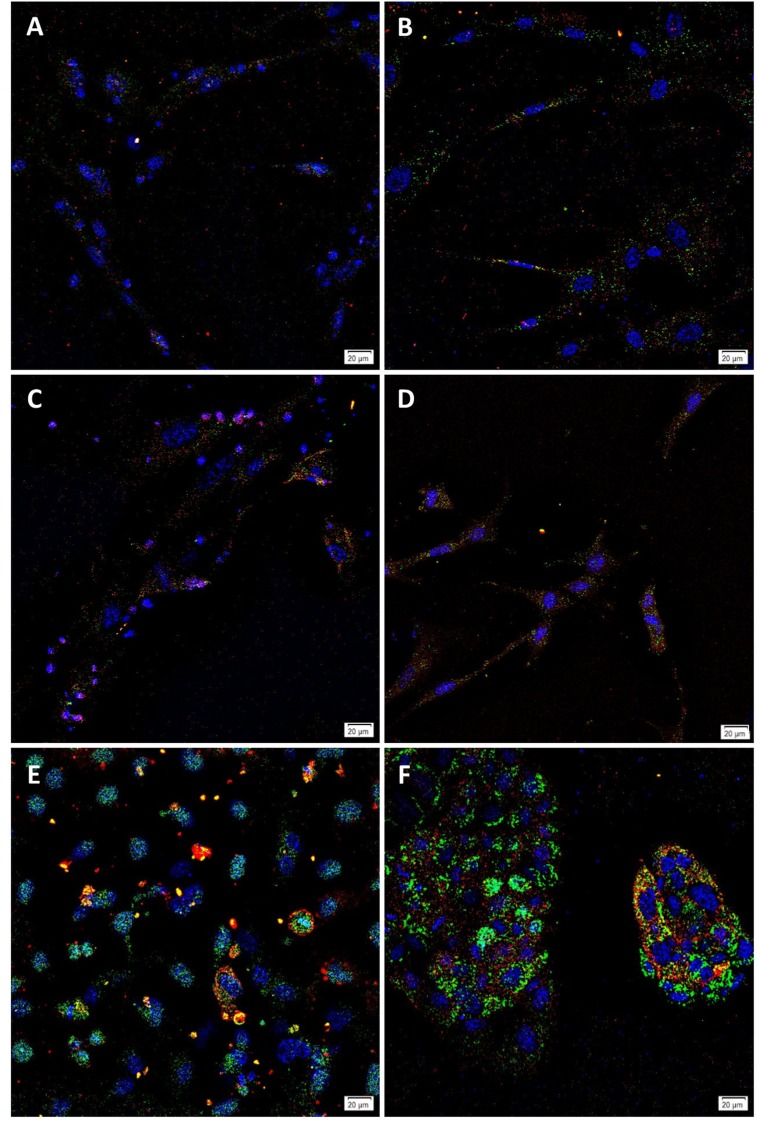
Representative immunofluorescence immunocytochemical images. EpCAM^Low^ (A) and EpCAM^High^ (B) cells from low-grade colon adenocarcinoma (LGCA)-derived primary cell lines, and EpCAM^Low^ (C) and EpCAM^High^ (D) cells from high-grade colon adenocarcinoma (HGCA)-derived primary cell lines showing expression of SOX2 (green) and TRA-1-60 (red). LGCA (n = 3); HGCA (n = 3). Positive control NTERA-2 (E) and CaCo2 (F) cells were stained for SOX2 (green) and TRA-1-60 (red) (B). Original magnification: 400x; scale bar = 20 μm.

SSEA4 (Fig 1A–1D) was expressed by all cell lines, and this was co-expressed with OCT4 in 2 of the 6 EpCAM^Low^ and 4 of the 6 EpCAM^High^ cell lines. Similarly, TRA-1-60 (Fig 2A–2D) was expressed in all cell lines, and this was co-expressed with SOX2 in 4 of the 6 EpCAM^Low^ and 5 of the 6 EpCAM^High^ cell lines.

Commercial cell lines NTERA-2 and CaCo2 were used as the positive controls for RT-qPCR and tumorsphere formation, respectively, and so their expression of key pluripotency markers was also assessed by ICC. Both cell lines contained cells which co-expressed each of the 4 PSC markers: OCT4 and SSEA4 (Fig 1E and 1F), and SOX2 and TRA-1-60 (Fig 2E and 2F).

### Tumorsphere formation assays

EpCAM^High^ and the EpCAM^Low^ cells derived from 3 low-grade CA (LGCA) and 3 high-grade CA (HGCA) tissue samples were cultured in ultra-low adherence plates with StemXVivo™ tumorsphere media. The threshold for positive tumorsphere formation was chosen to be an average diameter of 50 μm from at least 5 measured spheres per field of view [18–20]. A positive result was confirmed only if the tumorspheres could maintain their size until the time at which the cells in the centre of the tumorsphere began to necrose, seen as a dark centre, and the sphere began to break down.

**Fig 3:**
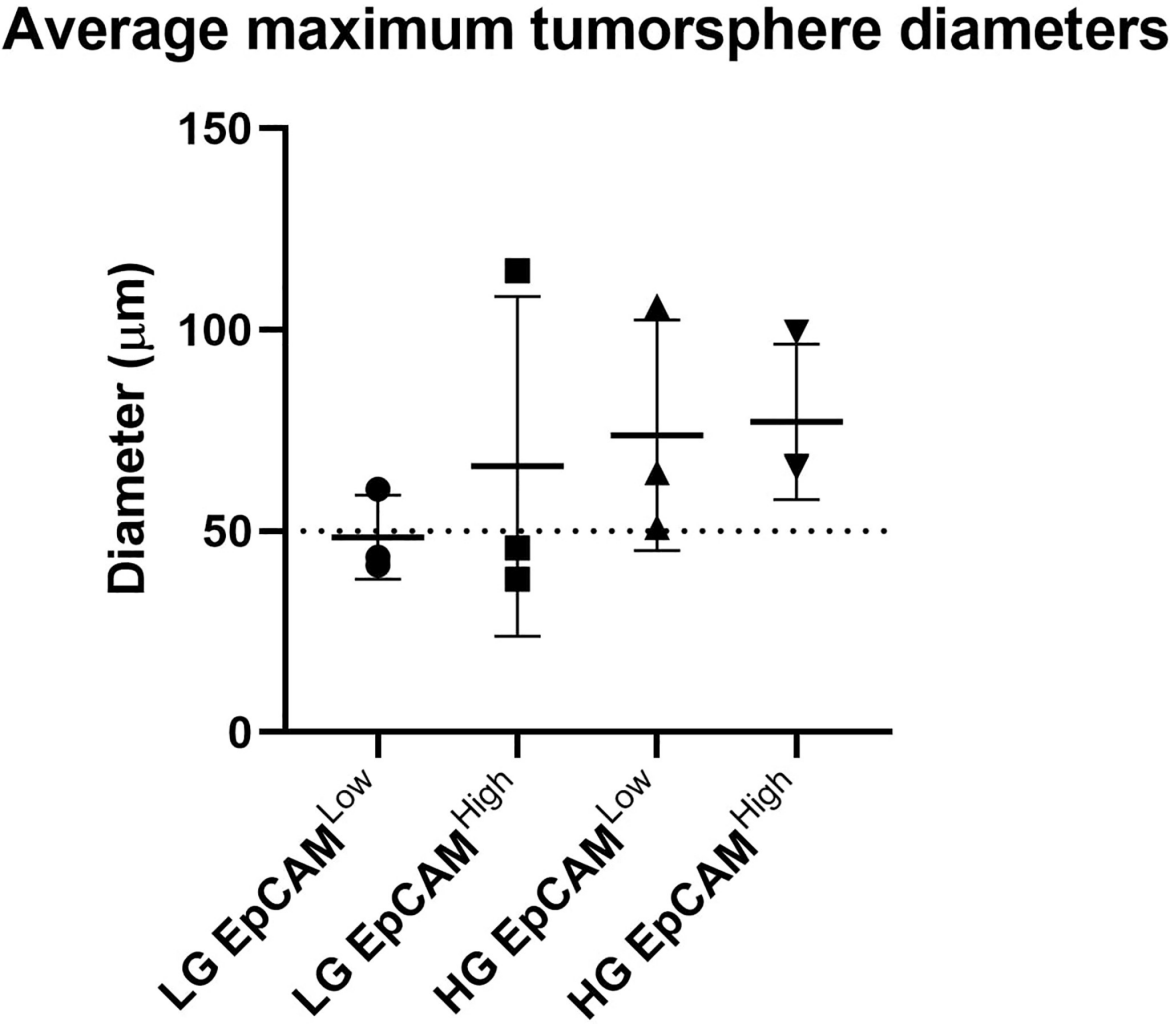
Maximum tumorsphere diameters for each condition. The average maximum tumorsphere diameter for each condition (LG EpCAM^Low^, LG EpCAM^High^, HG EpCAM^Low^ and HG EpCAM^High^) was calculated by combining the maximum diameter values from Table 1 for all technical and biological replicates per condition and calculating the average. Error bars show standard deviation.

The tumorsphere forming assay was carried out with three concurrent technical triplicates for each biological replicate (Fig 3). These results are displayed in Table 1, which shows that there was considerable variation between each of the biological replicates.

**Table 1:** Analysis of tumorsphere formation assay data.

Sample ID	Days to maximum diameter (average maximum diameter, μm)	Days at harvest (average diameter at harvest, μm)
LG_1 EpCAM^High^	4 (114.69)	6 (112.09)
LG_1 EpCAM^Low^	6 (60.48)	6 (60.48)
LG_2 EpCAM^High^	7 (38.00)	11 (33.00)
LG_2 EpCAM^Low^	10 (41.50)	14 (39.05)
LG_3 EpCAM^High^	8 (45.75)	15 (45.74)
LG_3 EpCAM^Low^	9 (43.50)	12 (43.50)
HG_1 EpCAM^High^	2 (99.46)	7 (95.54)
HG_1 EpCAM^Low^	1 (105.96)	7 (53.41)
HG_2 EpCAM^High^	6 (66.14)	8 (44.04)
HG_2 EpCAM^Low^	3 (50.95)	8 (48.00)
HG_3 EpCAM^High^	6 (65.85)	11 (57.88)
HG_3 EpCAM^Low^	5 (64.54)	11 (53.17)

Primary cell lines derived from 3 LGCA and 3 HGCA tissues samples were sorted into EpCAM^High^ and EpCAM^Low^ fractions. Tumorsphere diameter was measured in μm. Diameter values represent the average diameter of all measured tumorspheres across 3 technical replicates for each biological replicate.

**Fig 4:**
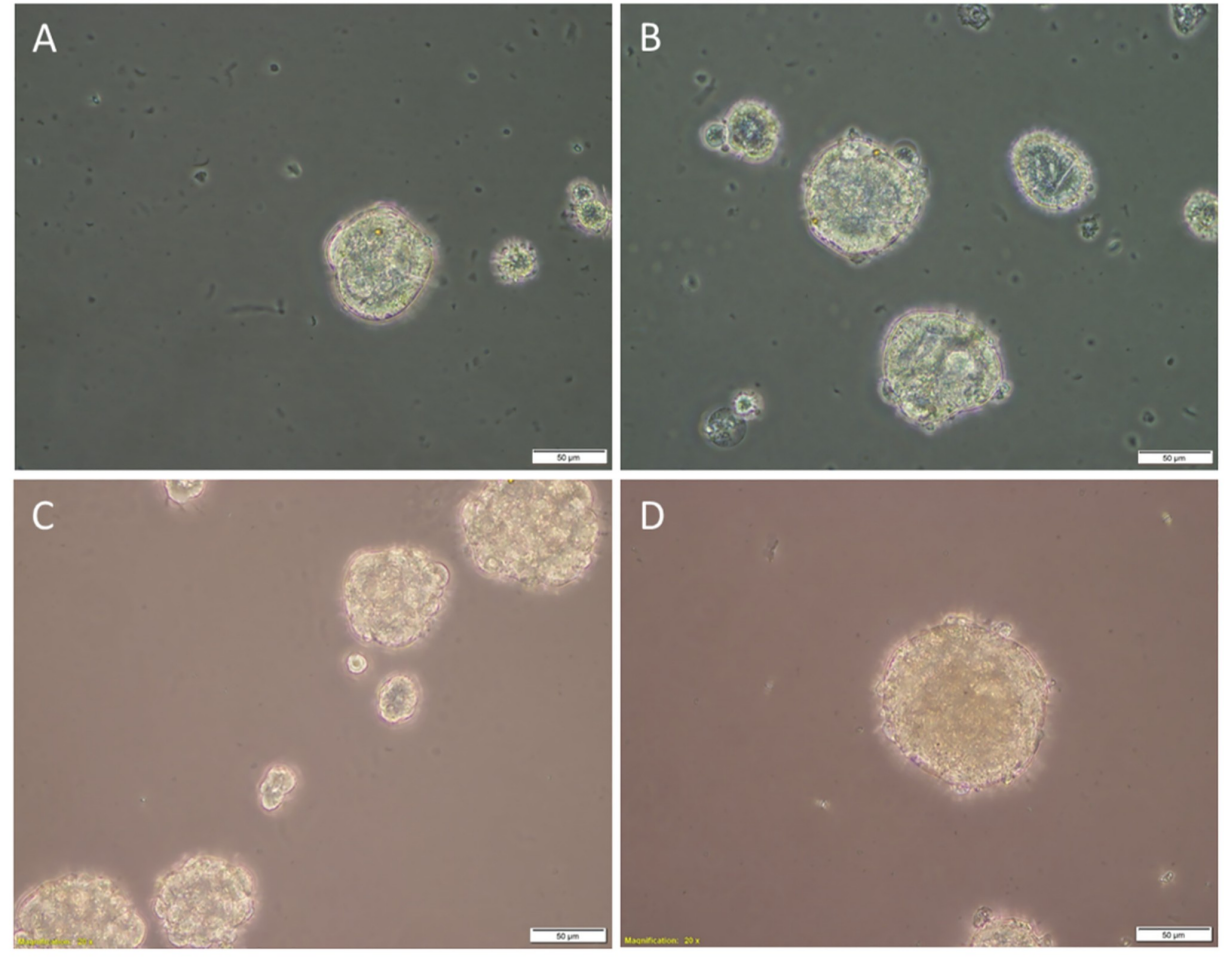
Representative images of tumorsphere formation assays. Tumorsphere formation in EpCAM^Low^ (A) and EpCAM^High^ (B) cells from low-grade colon adenocarcinoma (LGCA)-derived primary cell lines, and EpCAM^Low^ (C) and EpCAM^High^ (D) cells from high-grade colon adenocarcinoma (HGCA)-derived primary cell lines. LGCA (n = 3); HGCA (n = 3). Original magnification: 40x; scale bar = 50 μm.

Both the EpCAM^High^ and EpCAM^Low^ cells from all the 3 HGCA-derived primary cell lines attained a positive result for tumorsphere formation (Fig 4C and 4D).

The EpCAM^High^ and EpCAM^Low^ cells derived from the LG_1 sample (Fig 4A and 4B) formed tumorspheres (114.69 μm and 60.48 μm, respectively), but the EpCAM^High^ cells derived from LG_2 and the EpCAM^Low^ cells derived from LG_2 and LG_3 did not reach the size threshold (38.00 μm, 41.5 μm and 43.50 μm, respectively). Tumorspheres formed by LG_3-derived EpCAM^High^ cells were just below the diameter threshold (45.75 μm), and this was regarded as a negative result.

Interestingly, the EpCAM^High^ and the EpCAM^Low^ cells which were capable of forming tumorspheres reached the size threshold after a comparable number of days. However, the average size of the tumorspheres produced by EpCAM^High^ cells tended to be larger (71.7 μm after 5.5 d, n = 3) than those produced by EpCAM^Low^ cells (51.5 μm after 5.7 d, n = 3).

HGCA-derived cells produced tumorspheres which reached an average maximum diameter of 75.5 μm after an average of 3.8 d (EpCAM^High^ = 77.2 μm after 4.7 d, n = 3; EpCAM^Low^ = 73.8 μm after 3.0 d, n = 3).

In comparison, when LGCA cells were capable of producing tumorspheres, they reached an average maximum diameter of 48.7 μm after an average of 7.3 d (EpCAM^High^ = 66.2 μm after 6.3 d, n = 3; EpCAM^Low^ = 48.5 μm after 8.3 d, n = 3). Overall, both the EpCAM^High^ and EpCAM^Low^ cells from 1 of 3 LGCA and all the 3 HGCA were capable for forming tumorspheres which reached the size threshold.

Tumorspheres derived from 2 cell lines, 1 from a LGCA and the other from a HGCA, were passaged to confirm formation of true tumorspheres as opposed to cell clusters (Table 2). The EpCAM^High^ and EpCAM^Low^ cells derived from the LGCA sample both produced tumorspheres in their second passage. This took longer for the LGCA-derived EpCAM^Low^ cells in the second passage (53.3 μm after 7.0 d, n = 3) than in their first passage (60.5 μm after 6.0 d, n = 3).

**Table 2:** Data from passaged tumorspheres.

Sample ID	Days to maximum diameter (average diameter at maximum, μm)	Days at harvest (average diameter at harvest, μm)
LG_1 EpCAM^High^	9 (81.09)	14 (58.53)
LG_1 EpCAM^Low^	7 (53.29)	15 (35.91)
HG_1 EpCAM^High^	5 (53.02)	11 (45.05)
HG_1 EpCAM^Low^	6 (55.40)	11 (50.03)

Tumorspheres from 1 LGCA and 1 HGCA-derived primary cell line were passaged. Tumorspheres were isolated and separated into single cells to reform tumorspheres. Tumorsphere diameter was measured in μm. Diameter values represent the average diameter of all measured tumorspheres across three technical replicates for each biological replicate.

For the LGCA-derived EpCAM^High^ cells, the same observation was made and was even more pronounced: on average, tumorspheres reached a maximum diameter of 114.69 μm after 4 d in the first passage versus 81.09 μm after 9 d in the second passage.

The HGCA sample formed tumorspheres which reached the diameter threshold for both the EpCAM^High^ and EpCAM^Low^ cells (53.02 μm and 55.40 μm, respectively). However, this took considerably longer in the second passage (5 d and 6 d, respectively) than the first passage (2 d and 1 d, respectively).

### Differentiation assays

To further explore the stem cell functionality of these CA-derived cells, differentiation down the 3 embryonic germ lineages, mesoderm, endoderm and ectoderm, was induced.

**Fig 5:**
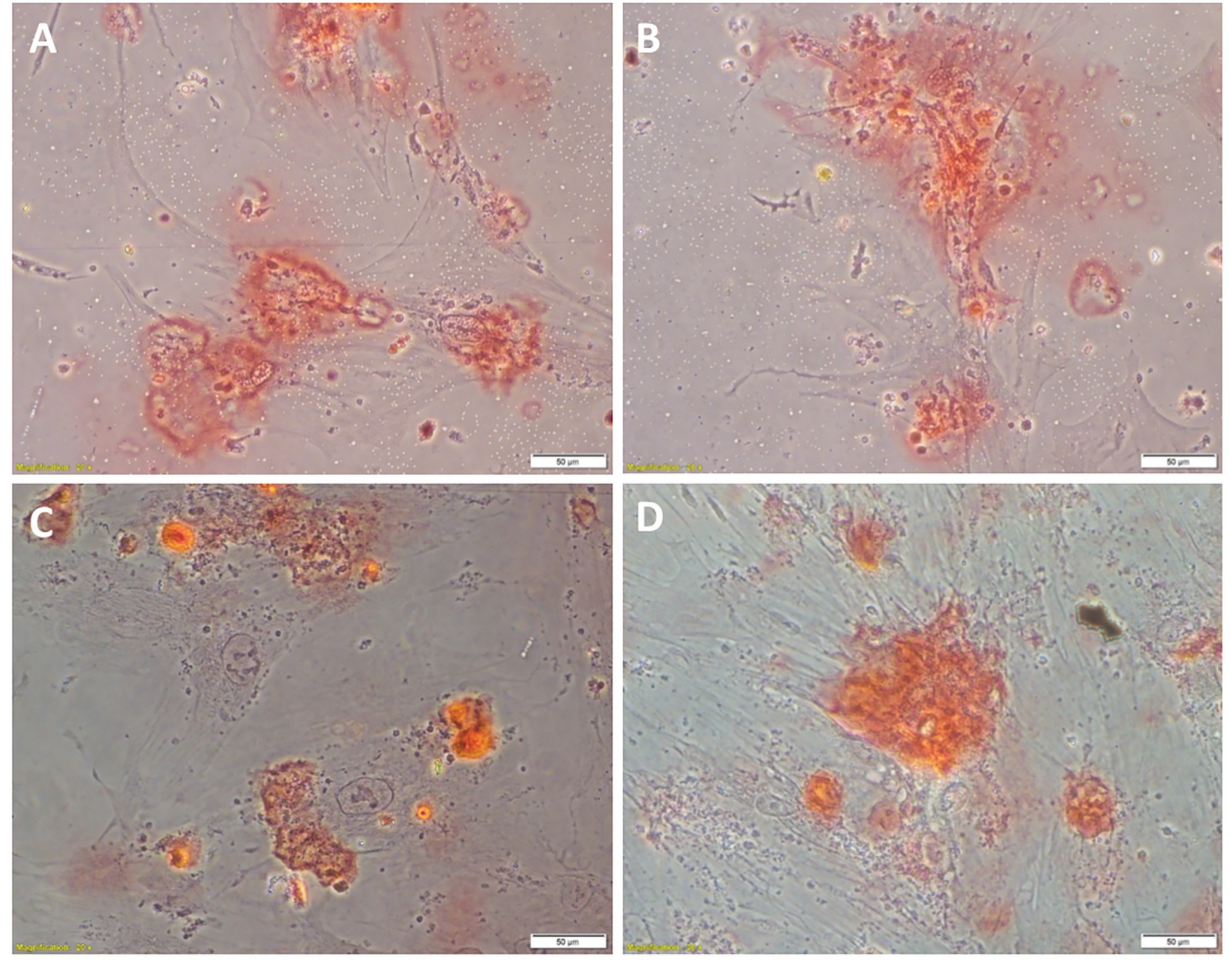
Representative images of Mesoderm differentiation. Primary tumor-derived EpCAM^Low^ (A) and EpCAM^High^ (B) LGCA cells, and EpCAM^Low^ (C) and EpCAM^High^ (D) HGCA cells, were grown in chambered slides for differentiation assays. Alizarin Red stain (pH 4.2) was used to detect calcium deposits. LGCA (n = 3); HGCA (n = 3). Original magnification = 40x; scale bar = 50 μm.

For mesodermal differentiation, cells were incubated in StemPro^®^ Osteogenesis media, and after up to 14 d in culture they were fixed and stained with Alizarin Red dye which binds specifically to calcium at pH 4.2. All the 3 LGCA and 3 HGCA cell lines showed positive staining to varying degrees (Fig 5), suggesting the capacity to differentiate down the mesodermal lineage.

**Fig 6:**
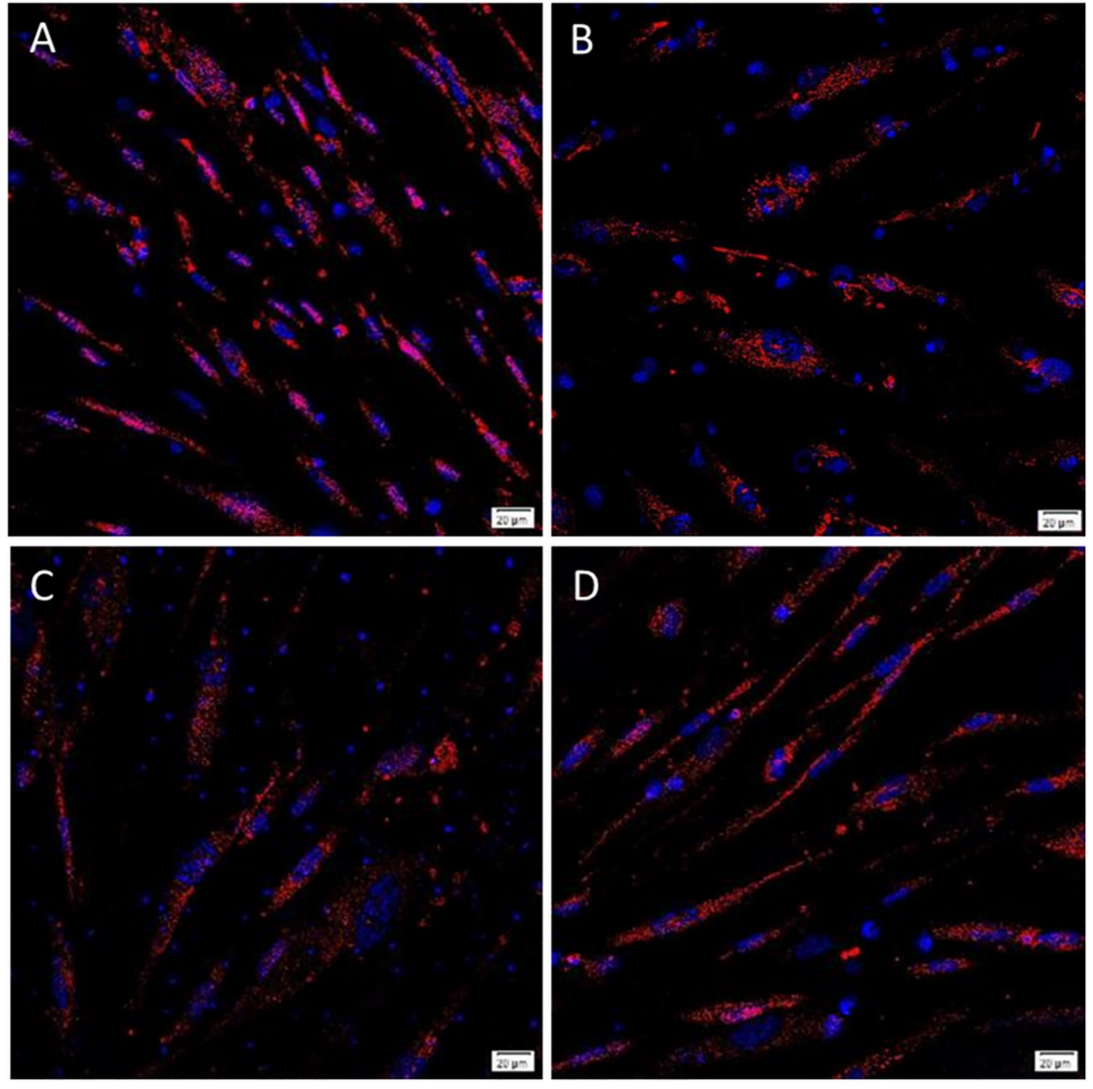
Representative images of Endoderm differentiation. Primary tumor-derived EpCAM^Low^ (A) and EpCAM^High^ (B) LGCA cells, and EpCAM^Low^ (C) and EpCAM^High^ (D) HGCA cells, were grown in chambered slides for differentiation assays. NorthernLights^™^ fluorescent secondary antibody (red) detected the rabbit anti-SOX17 primary antibody. LGCA (n = 3); HGCA (n = 3). Original magnification = 400x; scale bar = 20 μm.

Endodermal differentiation was induced using StemXVivo^®^ Endoderm media and confirmed using an antibody against SOX17. When imaged by confocal microscopy, all the 3 LGCA and 3 HGCA cell lines expressed SOX17 following incubation with the differentiation media, confirming the ability of these cells to differentiate down the endodermal lineage (Fig 6). These cells also expressed SOX17 when grown in regular culture media, but at much lower levels than in differentiation media (S12 Fig). This was consistent with the endodermal origins of colon.

**Fig 7:**
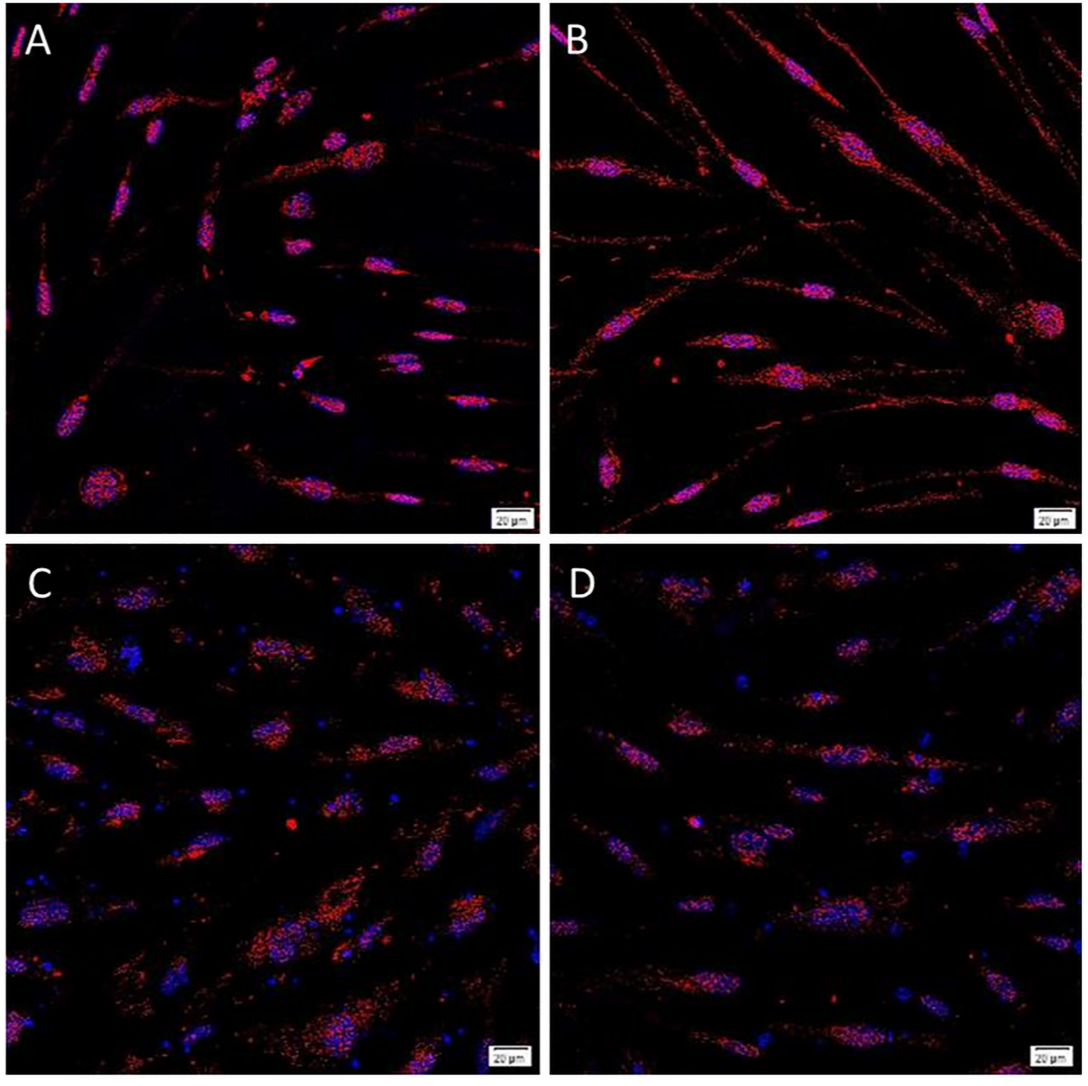
Representative images of Ectoderm differentiation. Primary tumor-derived EpCAM^Low^ (A) and EpCAM^High^ (B) LGCA cells, and EpCAM^Low^ (C) and EpCAM^High^ (D) HGCA cells, were grown in chambered slides for differentiation assays. NorthernLights™ fluorescent secondary antibody (red) detected the rabbit anti-Otx2 primary antibody. LGCA (n = 3); HGCA (n = 3). Original magnification = 400x; scale bar = 20 μm.

The StemXVivo^®^ Ectoderm kit was used to induce ectodermal differentiation, using Otx2 as an ectodermal marker. Surprisingly, all control cells grown in regular media (S15 Fig) expressed Otx2 at similar levels to the cells incubated in the differentiation media (Fig 7).

### RT-qPCR

**Fig 8:**
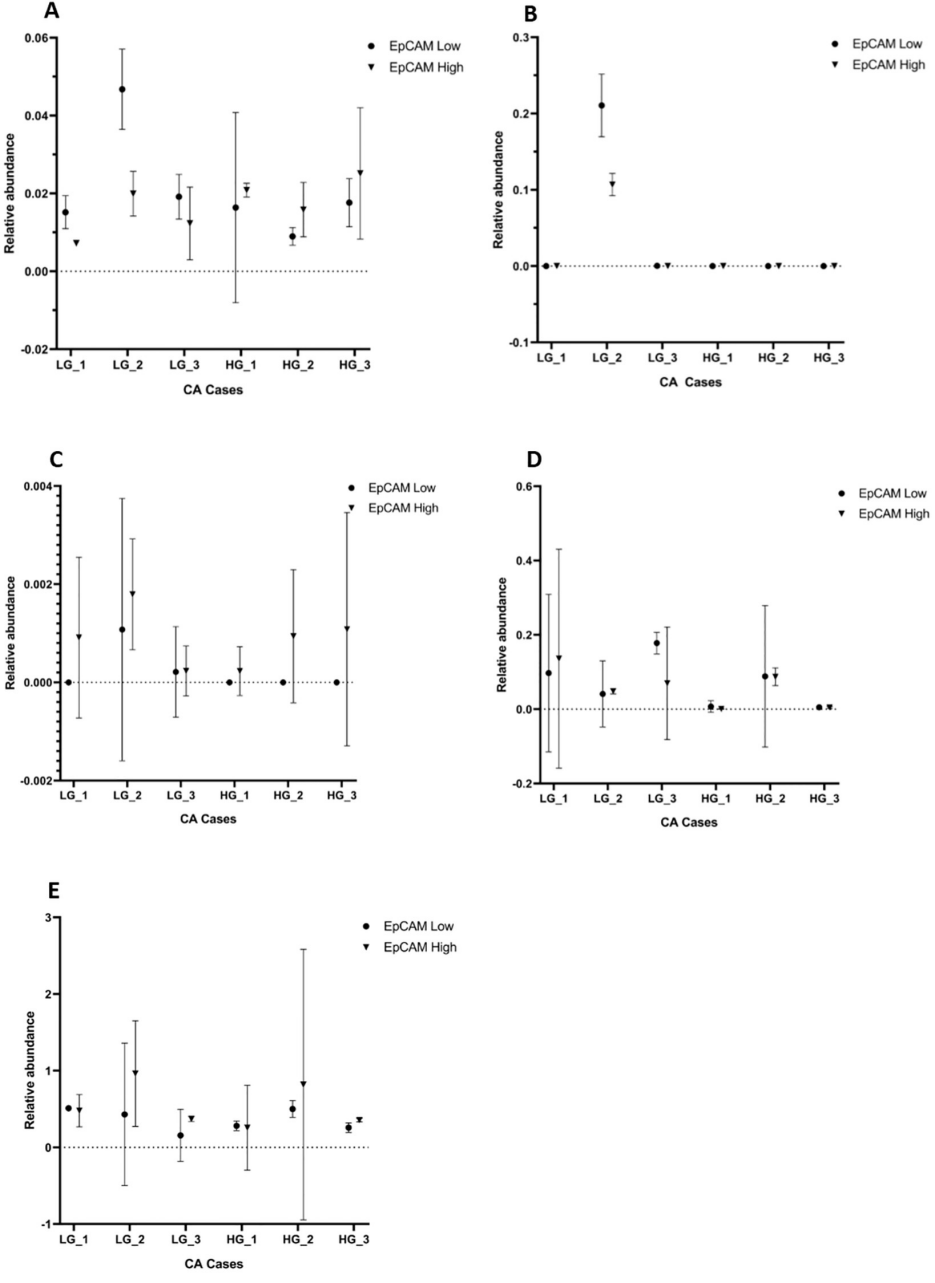
RT-qPCR. RNA was extracted from EpCAM^High^ and EpCAM^Low^ cells lines from 3 LGCA and 3 HGCA cases, and RT-qPCR was carried out to measure the mRNA levels of OCT4 (A), SOX2 (B), NANOG (C), KLF4 (D) and c-MYC (E). Triplicate values are shown by dots (EpCAM^Low^) and squares (EpCAM^High^), with mean and 95% confidence intervals. Abundance was measured relative to qPCR Human Reference Total RNA (Mediray). LGCA (n = 3); HGCA (n = 3).

To investigate mRNA expression of iPSC genes, RNA was extracted from cells and analyzed by RT-qPCR (Fig 8). OCT4 mRNA was detected in all CA-derived primary cell lines and was found to be more abundant in the EpCAM^Low^ cells than the EpCAM^High^ cells derived from all the 3 LGCA samples. However, it was more abundant in the EpCAM^High^ cells than the EpCAM^Low^ cells derived from all the 3 HGCA samples. NANOG was detected in EpCAM^High^ cells derived from all the 3 LGCA samples and EpCAM^Low^ and EpCAM^High^ cells from all the 3 HGCA samples, but was only present in EpCAM^Low^ cells from 1 of the the 3 LGCA-derived primary cell lines. SOX2 mRNA was present in 2 EpCAM^Low^ and 1 EpCAM^High^ LGCA-derived cell lines, but it was below the detection threshold in all the 3 HGCA-derived cell lines. KLF4 mRNA was more abundant in LGCA-derived cells than HGCA-derived cells, with detection above the threshold in all EpCAM^Low^ cells and all but 1 HGCA EpCAM^High^ cell lines.

Of the 5 iPSC genes, c-MYC was the most highly expressed and was seen in all EpCAM^Low^ and EpCAM^High^ cells, with higher expression in EpCAM^High^ cells relative to their EpCAM^Low^ counterparts.

### Western blotting

**Fig 9:**
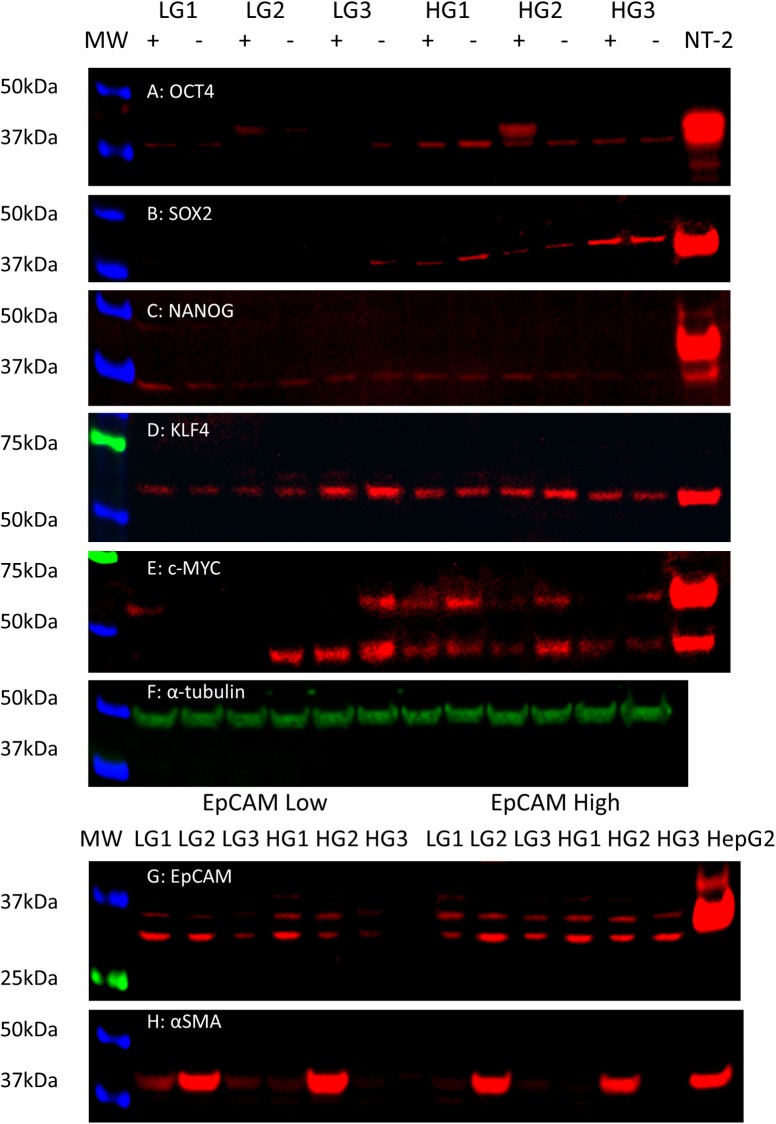
Western blotting. Protein extractions from 3 LGCA and 3 HGCA EpCAM^High^ (+) and EpCAM^Low^ (-) cell lines were probed for OCT4 (A; 40kDa), SOX2 (B; 40-43kDa), NANOG (C; 37-40kDa), KLF4 (D; 54kDa) and c-MYC (E; 42 & 57kDa). NTERA-2 cells were used as the positive control for all iPSC markers. α-tubulin (F; 50kDa) was used as a loading control. EpCAM^High^ and EpCAM^Low^ cell lines were also probed for their expression of EpCAM (G; bands from ~30-40kDa) and α-SMA (H; 42kDa). HepG2 cells and 3T3 cells were used as the positive control for EpCAM and α-SMA, respectively.

**Fig 10:**
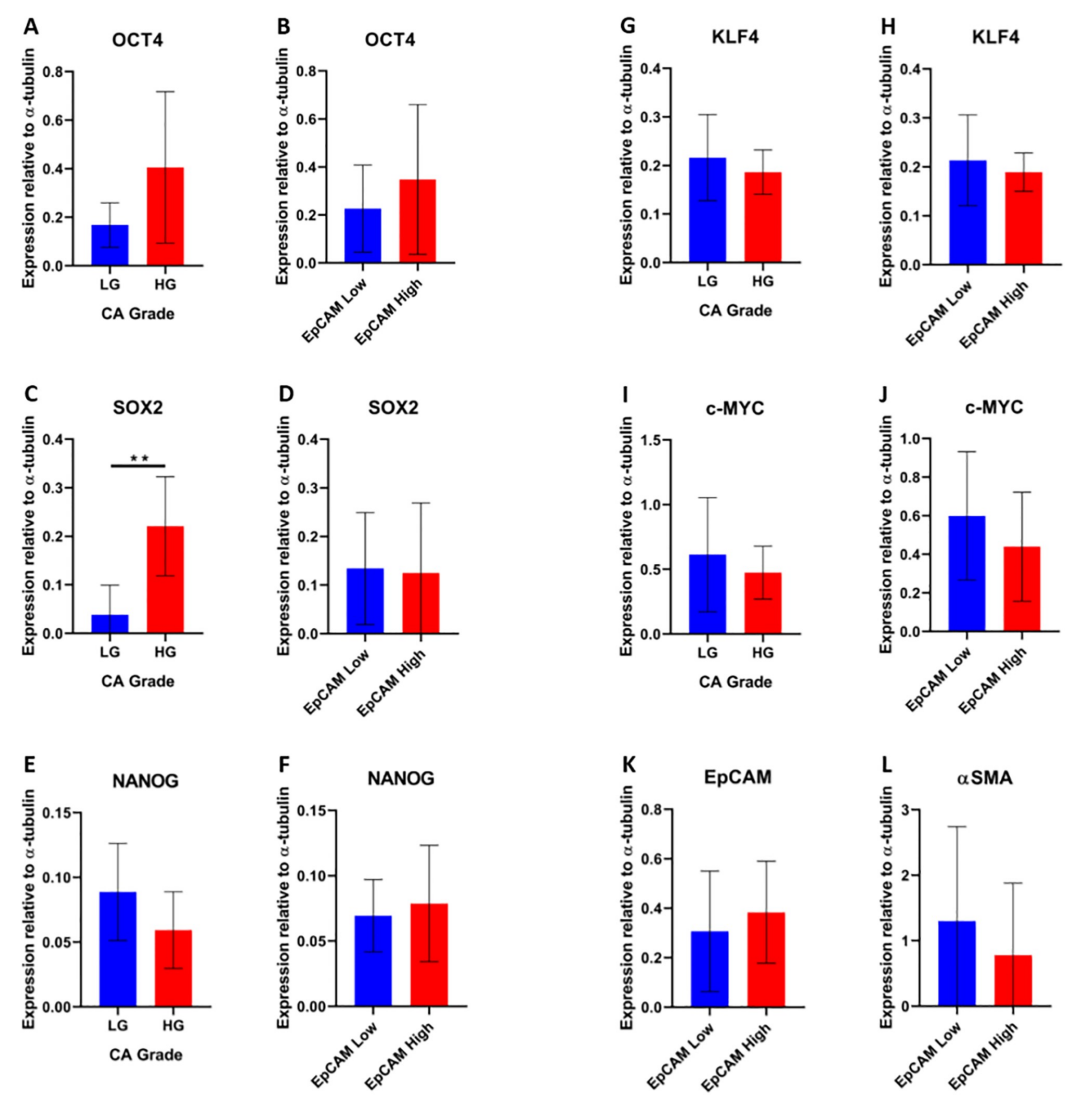
Densitometry performed on western blot. Densitometry data provided semi-quantitative data for protein abundance. The intensity values of all LGCA cell lines (both EpCAM^High^ and EpCAM^Low^) and all HGCA cell lines (both EpCAM^High^ and EpCAM^Low^) were combined and the average intensity calculated, and these are shown for OCT4 (A), SOX2 (C), NANOG (E), KLF4 (G) and c-MYC (I). The intensity values of all EpCAM^Low^ cell lines (both LGCA and HGCA) and all EpCAM^High^ cell lines (both LGCA and HGCA) were combined and the average intensity calculated, and these are shown for OCT4 (B), SOX2 (D), NANOG (F), KLF4 (H), c-MYC (J), EpCAM (K) and α-SMA (L). Individual intensity values were normalized against the loading control α-tubulin before being combined and averaged. Error bars show standard deviation.

The protein products of the 5 iPSC genes were investigated by WB (Fig 9A–9E) and analyzed semi-quantitatively using densitometry (Fig 10).

OCT4, SOX2, KLF4 and c-MYC proteins were detected in both the EpCAM^High^ and EpCAM^Low^ cells from all the 3 HGCA-derived cell lines.

OCT4 (Figs 9A and 10A) was present in the EpCAM^High^ and EpCAM^Low^ cells derived from 2 of the LGCA samples, and only the EpCAM^Low^ cells derived from the third sample. Similarly, SOX2 (Figs 9B and 10C) was detected in the EpCAM^Low^ cells derived from 1 LGCA sample, but it was undetectable in the other 2 LGCA samples. Very faint bands corresponding to the NANOG protein (Figs 9C and 10E) were seen in EpCAM^High^ cells derived from 2 LGCA and 2 HGCA samples, and EpCAM^Low^ cells derived from three LGCA and 2 HGCA samples. KLF4 (Figs 9D and 10G) was the only marker to be detected in both the EpCAM^High^ and EpCAM^Low^ cells from all LGCA and HGCA samples. c-MYC (Figs 9E and 10I) was present in the EpCAM^High^ and EpCAM^Low^ cells derived from one LGCA sample. Of the other 2 LGCA samples, c-MYC was only expressed in EpCAM^High^ cells derived from 1 sample and only in the EpCAM^Low^ cells derived from the other sample. Overall, there was a greater amount of c-MYC protein found in EpCAM^Low^ cells than EpCAM^High^ cells.

When comparing the levels of marker expression between LG and HG cell lines, only SOX2 showed a statistically significant increase (p = 0.0037). The difference in OCT4 expression between LG and HG cell lines was noticeable but was just below statistical significance (p = 0.07). There were no statistically significant differences in marker expression between EpCAM^High^ and EpCAM^Low^ cells (Fig 10B, 10D, 10F, 10H and 10J).

To assess the efficiency of the EpCAM sort and the characteristics of the resulting subpopulations of cells, protein extracts from all 6 EpCAM^Low^ and 6 EpCAM^High^ cell lines were probed for EpCAM (Fig 9G) and α-SMA (Fig 9H). All of the cells expressed EpCAM, however, the corresponding bands for EpCAM were noticeably stronger in the EpCAM^High^ cells than the EpCAM^Low^ cells when assessed using densitometry (Fig 10K). The observed bands are thought to correspond to un-glycosylated EpEX (the extracellular domain of EpCAM; 30kDa), glycosylated EpEX and un-glycosylated full-length EpCAM (~35kDa), and glycosylated full-length EpCAM (40kDa in HepG2). The same cells were then probed using an antibody against α-SMA, a stromal marker, to see whether there might be an inverse relationship between expression levels of EpCAM and α-SMA. This appeared to be the case, with stronger staining for α-SMA in EpCAM^Low^ cells than in EpCAM^High^ cells (Fig 10L).

## Discussion

Based on our earlier work demonstrating the expression of iPSC markers OCT4, SOX2, NANOG, KLF4 and c-MYC by subpopulations of CSCs in primary LCGA and HGCA tissue samples [16], this study assessed the stem cell characteristics of primary cell lines derived from some of these tissue samples using tumorsphere formation and differentiation assays.

When induced to differentiate down the three embryonic lineages, these CA-derived cells were capable of undergoing endodermal, ectodermal and mesodermal differentiation, as evidenced by their expression of SOX17 and Otx2, and formation of calcium deposits, respectively. This was validated by comparing with negative controls where the cells were grown in their regular culture media, as well as with the omission of the primary antibody.

However, somewhat unexpectedly, cells were found to be widely positive for Otx2 when grown in both the ectoderm differentiation media and regular media. Notably, there have been some concerns regarding the specificity of Otx2 as a marker for ectoderm [21]. The Human Protein Atlas suggests Otx2 is expressed weakly in the colon, specifically by the goblet cells. There is also some evidence suggesting Otx2 is expressed by cells within the renal tubules, bile ducts and seminiferous ducts of the testis, and neuronal and glial cells, as highlighted by the Human Protein Atlas. Despite being used as the marker for ectodermal differentiation in this kit, it may be beneficial to find a more specific marker for ectoderm.

In our previous study [16] we assessed the expression pattern of EpCAM in normal colon and CA tissues using IHC staining, which revealed that EpCAM expression is restricted to epithelial cells in our normal colon and CA tissues. Therefore, for this study we sought to culture cells derived from these tissues and then isolate the epithelial and stromal subpopulations by sorting using an anti-EpCAM antibody bound to magnetic beads. After 2 passages post-sorting to allow cells to recover, total protein was extracted from EpCAM^High^ and EpCAM^Low^ cells from all CA-derived primary cell lines for WB. When probing for EpCAM, bands were detected at the expected molecular weight in EpCAM^High^ cells, but also in the EpCAM^Low^ cells. Furthermore, when WB was performed for the stromal marker α-SMA, it was observed that both EpCAM^Low^ and EpCAM^High^ populations expressed α-SMA. However, EpCAM expression was higher in EpCAM^High^ cells than in EpCAM^Low^, and α-SMA expression was higher in EpCAM^Low^ cells than in EpCAM^High^. This suggests that there is some difference in phenotype between the two subpopulations resulting from the EpCAM sort.

When growing a primary cell line from a tissue sample, it has been observed that the expression of up to 10% of genes is altered within five passages [22]. In general, there is a selection pressure which favors an adherent and proliferative phenotype [23]. The expression profile of these cells tends to drive them towards a more robust and stem-like phenotype [23]. In fact, it has been widely observed that cells derived from different tissues all become more similar to each other when grown in culture [24, 25]. Furthermore, Sandberg and Ernberg [26] have shown that when gene expression is analyzed, cancer tissue samples are more similar to their patient-matched normal tissue samples than to the primary cell strains derived from them. Amongst the genes most commonly upregulated in cell culture relative to their primary tissue source are the adhesion molecules, which may suggest an emergence of EpCAM expression in our EpCAM^Low^ sorted cells [25]. Alternatively, because the cells had been in culture for 7–10 passages at the time of sorting, it may be that any EpCAM-negative cells present when establishing the primary cell culture were outcompeted or had acquired EpCAM expression before sorting. This raises the possibility that most of the cells were already expressing EpCAM and the antibodies on the beads became saturated, leaving some EpCAM positive (or possibly EpCAM^Low^) cells to be collected in the EpCAM “negative” fraction.

The method of establishing cell lines before banking may contribute to selection for certain cell phenotypes. The Gillies McIndoe Research Institute tissue bank uses an explant method, in which a small piece of tumor tissue is embedded in Matrigel and cells migrate from the tissue into the matrix. Following this, the matrix and tissue piece are dissociated using trypsin and pelleted by centrifugation. A range of cell types are present when the pellet is transferred to a culture flask, including red blood cells which are eliminated after the first passage, suggesting that multiple tumor cell types will be present. However, this method of procurement may result preferentially in a cell line with a migratory and possibly proliferative phenotype, at the expense of other cell types, thus not fully reflecting the cellular heterogeneity of the original tumor tissue. This potential limitation was taken into account when analyzing our results, and is a subject of future work.

There are two important points to be considered in this regard. Firstly, observations of altered gene expression do not necessarily mean that expression or regulation at the protein level has changed [22]. Secondly, cell culture can be a valuable experimental tool, although when designing an experiment, the effects of cell culture on gene and protein expression should be considered. These concerns may be mitigated by using cells with a very low passage number for functional work whenever possible, or by using tissue samples when available for assays such as RT-qPCR and WB.

Further evidence of the influence of EpCAM expression was seen through the ICC PSC marker assays and tumorsphere formation assays carried out in this study. The ICC PSC kit was employed as a standardized way to assess the expression of validated pluripotency markers [27–31]. Validated pluripotency markers SSEA4 and TRA-1-60 were expressed by a subset of cells in all cell lines assayed, which co-expressed with OCT4 in 2 of the 6 EpCAM^Low^ and 4 of the 6 EpCAM^High^ cell lines and with SOX2 in 4 of the 6 EpCAM^Low^ and 5 of the 6 EpCAM^High^ cell lines. This mirrors our results in CA tissues [16], where SOX2 was more abundant than both OCT4 and NANOG, co-localizing to both the OCT4^+^ and NANOG^+^ CSC subpopulations. The co-expression of OCT4, SOX2, SSEA4 and TRA-1-60 suggests that a subpopulation of stem-like cells is present within these cultured cell strains. There seemed to be a noticeable difference between EpCAM^High^ and EpcAM^Low^ cells in terms of OCT4 expression, which was seen in 4 of the 6 EpCAM^High^ cells lines but only in 2 of the 6 EpCAM^Low^ cell lines. However, this differed from the results of tissue samples analyzed [16] in which OCT4 was expressed in the tumor stroma by EpCAM-negative cells. This may indicate selection for or drive towards a stem-like phenotype and similarities in gene expression in culture, whereby EpCAM^-^ cells may begin to express EpCAM, or EpCAM^+^ cells begin to express OCT4. Interestingly, OCT4 mRNA levels were higher in LGCA-derived EpCAM^Low^ cells than EpCAM^High^ cells, a result which is consistent with our experiments in tissues, but lower in HGCA-derived EpCAM^Low^ cells than EpCAM^High^ cells.

Furthermore, in the tumorsphere formation assays, EpCAM^High^ cells consistently performed better than EpCAM^Low^ cells, suggesting a functional difference between these two subpopulations, possibly due to the expression of EpCAM and the correlation between EpCAM and iPSC marker levels. When the average diameters were calculated across the 3 biological replicates from each condition group, the size of tumorspheres produced by each condition increased in the order of LCGA EpCAM^Low^, LGCA EpCAM^High^, HGCA EpCAM^Low^, and HGCA EpCAM^High^ (Fig 3). To account for the variability seen in this assay, and to see whether statistical significance can be achieved, we intend to repeat these experiments at a later date once we have collected more primary cell lines.

The results in Table 1 show that there is variation between each of the biological replicates. The size of tumorspheres produced by each cell line was relatively proportional to their vigor of growth as a monolayer in culture, and indeed reflects the inherent heterogeneity which exists between tumors from different individuals. Whilst each sample is graded as HG or LG for convenience, in reality tumors are stratified on an arbitrary scale which takes into account a wide range of factors, and all cases of a given grade will be different to each other in various histological characteristics. However, we can observe trends at the population level, as displayed in Fig 3.

The functional ability of HGCA-derived primary cell lines to form tumorspheres of a larger size than LGCA-derived cells mirrors the WB results, which showed that HGCA-derived cells expressed higher levels of OCT4, SOX2, NANOG and c-MYC proteins than LGCA-derived cells. The iPSC markers, especially OCT4, SOX2 and NANOG, are linked to stem-like characteristics such as tumorsphere formation and maintenance of pluripotency [9, 10, 32], and so it was not surprising to find that the cells which produce larger tumorspheres have higher expression of iPSC markers. It has been suggested that KLF4 expression is inversely correlated with CA tumor grade, with HGCA producing less KLF4 than LGCA tumors, and the highest expression seen in the normal colon adjacent to tumors [15]. This was also demonstrated in our PCR results, where two HGCA samples had significantly lower levels of KLF4 mRNA than all the 3 of the LGCA samples. WB showed that protein abundance was relatively similar in LGCA and HGCA cell lines. Another interesting example of this is the LG_2 sample, which when compared to the other two LGCA samples had higher mRNA expression for OCT4, SOX2 and NANOG and much lower levels of KLF4 mRNA, perhaps placing it somewhere near the boundary between low-grade and high-grade. As we have previously addressed [16], there is a well-documented discrepancy between SOX2 mRNA levels and protein levels. SOX2 mRNA abundance is in almost every case much lower than the protein levels [9, 33], which we also observed in these cell lines.

Due to the financial and time costs associated with animal studies, as well as the ethical implications of such work, alternatives to xenograft and teratoma experiments in animals is the focus of much review [34]. While they remain valuable and perhaps essential for applications such as safety testing of stem cell therapies, there are a range of *in vitro* tests for assessing pluripotency that negate the need for animal testing. Teratoma assay protocols are often vague and inconsistent, and are not highly standardized and reproducible [34]. To determine whether a cell population includes pluripotent cells, it is sufficient to employ directed or spontaneous differentiation, tumorsphere formation which can be sustained over multiple passages, and an analysis of pluripotency marker expression [34]. It has become more acceptable to use markers including OCT4, SOX2, NANOG, SSEA4 and TRA-1-60 to identify cells which are pluripotent [5, 7, 8, 35]. Our assessment of pluripotency in subpopulations of CA-derived cells is in line with this approach and we plan to extend this work with further characterization using a wider panel of validated stem cell markers.

In conclusion, these experiments demonstrated that primary cell lines derived from LGCA and HGCA tissue samples are capable of forming tumorspheres which can be recapitulated upon passaging, and can differentiate down the three embryonic lineages, supporting the presence of CSC-like pluripotent subpopulations which we have previously identified in CA tissue samples. The observation of iPSC markers having distinct expression profiles in HGCA-derived and LGCA-derived primary cell lines indicates that it may be possible to use iPSC markers in a prognostic context and to aid in grading of these tumors. These findings build on the results of our earlier work on CA tissue samples [16].

## Supporting information

S1 Fig Representative images of immunofluorescence immunocytochemical negative controls. Negative controls were run for the control cells by omitting the primary antibodies for SSEA4/OCT4 (A) and for SOX2/TRA-1-60 (B) in NTERA-2 cells, and for SSEA4/OCT4 (C) and for SOX2/TRA-1-60 (D) in CaCo2 cells. Original magnification = 400x; scale bar = 20 μm.
(TIF)

S2 Fig Representative images of immunofluorescence immunocytochemical staining for sample LG1. EpCAM^Low^ cells from sample LG1 were stained for SSEA4 (green; A) and OCT4 (red; A), and for SOX2 (green; C) and TRA-1-60 (red; C). EpCAM^High^ cells from sample LG1 were stained for SSEA4 (green; B) and OCT4 (red; B), and for SOX2 (green; D) and TRA-1-60 (red; D). Original magnification = 400x; scale bar = 20 μm.
(TIF)

S3 Fig Representative images of immunofluorescence immunocytochemical staining for sample LG2. EpCAM^Low^ cells from sample LG2 were stained for SSEA4 (green; A) and OCT4 (red; A), and for SOX2 (green; C) and TRA-1-60 (red; C). EpCAM^High^ cells from sample LG2 were stained for SSEA4 (green; B) and OCT4 (red; B), and for SOX2 (green; D) and TRA-1-60 (red; D). Original magnification = 400x; scale bar = 20 μm.
(TIF)

S4 Fig Representative images of immunofluorescence immunocytochemical staining for sample LG3. EpCAM^Low^ cells from sample LG3 were stained for SSEA4 (green; A) and OCT4 (red; A), and for SOX2 (green; C) and TRA-1-60 (red; C). EpCAM^High^ cells from sample LG3 were stained for SSEA4 (green; B) and OCT4 (red; B), and for SOX2 (green; D) and TRA-1-60 (red; D). Original magnification = 400x; scale bar = 20 μm.
(TIF)

S5 Fig Representative images of immunofluorescence immunocytochemical staining for sample HG1. EpCAM^Low^ cells from sample HG1 were stained for SSEA4 (green; A) and OCT4 (red; A), and for SOX2 (green; C) and TRA-1-60 (red; C). EpCAM^High^ cells from sample HG1 were stained for SSEA4 (green; B) and OCT4 (red; B), and for SOX2 (green; D) and TRA-1-60 (red; D). Original magnification = 400x; scale bar = 20 μm.
(TIF)

S6 Fig Representative images of immunofluorescence immunocytochemical staining for sample HG2. EpCAM^Low^ cells from sample HG2 were stained for SSEA4 (green; A) and OCT4 (red; A), and for SOX2 (green; C) and TRA-1-60 (red; C). EpCAM^High^ cells from sample HG2 were stained for SSEA4 (green; B) and OCT4 (red; B), and for SOX2 (green; D) and TRA-1-60 (red; D). Original magnification = 400x; scale bar = 20 μm.
(TIF)

S7 Fig Representative images of immunofluorescence immunocytochemical staining for sample HG3. EpCAM^Low^ cells from sample HG3 were stained for SSEA4 (green; A) and OCT4 (red; A), and for SOX2 (green; C) and TRA-1-60 (red; C). EpCAM^High^ cells from sample HG3 were stained for SSEA4 (green; B) and OCT4 (red; B), and for SOX2 (green; D) and TRA-1-60 (red; D). Original magnification = 400x; scale bar = 20 μm.
(TIF)

S8 Fig Representative images of tumoursphere formation assay positive controls. CaCo2 cells were used as a positive control for tumorsphere formation assays, here showing tumorsphere formation at day 7. Original magnification = 100x; scale bar = 200 μm.
(TIF)

S9 Fig Mesoderm differentiation positive controls. 3T3 cells (A) and CaCo2 cells (B) were used as positive controls for mesodermal differentiation. Negative controls were run by growing 3T3 cell (C) and CaCo2 cells (D) in regular culture media rather than differentiation media. Original magnification = 40x; scale bar = 50 μm.
(TIF)

S10 Fig Mesoderm differentiation negative controls. As a negative control, primary tumor-derived EpCAM^Low^ (A) and EpCAM^High^ (B) LGCA cells, and EpCAM^Low^ (C) and EpCAM^High^ (D) HGCA cells, were grown in regular culture media rather than differentiation media before being exposed to Alizarin Red (pH4.2). Original magnification = 40x; scale bar = 50 μm.
(TIF)

S11 Fig Endoderm differentiation negative controls. Negative controls were run for EpCAM^Low^ (A) and EpCAM^High^ (B) LGCA cells, and EpCAM^Low^ (C) and EpCAM^High^ (D) HGCA cells by omitting the anti-SOX17 primary antibody. Original magnification = 400x; scale bar = 20 μm.
(TIF)

S12 Fig Endoderm differentiation negative controls. Negative controls were run for EpCAM^Low^ (A) and EpCAM^High^ (B) LGCA cells, and EpCAM^Low^ (C) and EpCAM^High^ (D) HGCA cells by growing them in regular culture media rather than differentiation media before being exposed to anti-SOX17. Original magnification = 400x; scale bar = 20 μm.
(TIF)

S13 Fig Endoderm differentiation controls. CaCo2 cells were used as a positive control for endoderm differentiation (A). CaCo2 were also grown in regular culture media as a control (B and C). A negative control was run by omitting the anti-SOX17 primary antibody from CaCo2 cells grown in differentiation media (D). Original magnification = 400x; scale bar = 20 μm.
(TIF)

S14 Fig Ectoderm differentiation negative controls. Negative controls were run for EpCAM^Low^ (A) and EpCAM^High^ (B) LGCA cells, and EpCAM^Low^ (C) and EpCAM^High^ (D) HGCA cells by omitting the anti-Otx2 primary antibody. Original magnification = 400x; scale bar = 20 μm.
(TIF)

S15 Fig Ectoderm differentiation negative controls. Negative controls were run for EpCAM^Low^ (A) and EpCAM^High^ (B) LGCA cells, and EpCAM^Low^ (C) and EpCAM^High^ (D) HGCA cells by growing them in regular culture media rather than differentiation media before being exposed to anti-Otx2. Original magnification = 400x; scale bar = 20 μm.
(TIF)

S16 Fig Ectoderm differentiation controls. CaCo2 cells were used as a positive control for ectoderm differentiation (A). CaCo2 were also grown in regular culture media as a control (B). A negative control was run by omitting the anti-Otx2 primary antibody from CaCo2 cells grown in differentiation media (C). Original magnification = 400x; scale bar = 20 μm.
(TIF)

S17 Fig Full unmodified western blotting images. Uncropped images of western blotting membranes before adjusting for background fluorescence, showing OCT4 (A), SOX2 (B), NANOG (C), KLF4 (D), c-MYC (E), α-tubulin (F), EpCAM (G) and α-SMA (H).
(TIF)
