# Supplementary figures and images for: Colon adenocarcinoma-derived cells that express induced-pluripotent stem cell markers possess stem cell function
Source: PLoS One. 2020 May 19;15(5):e0232934. doi: 10.1371/journal.pone.0232934 (PMC7236985; doi:10.1371/journal.pone.0232934)

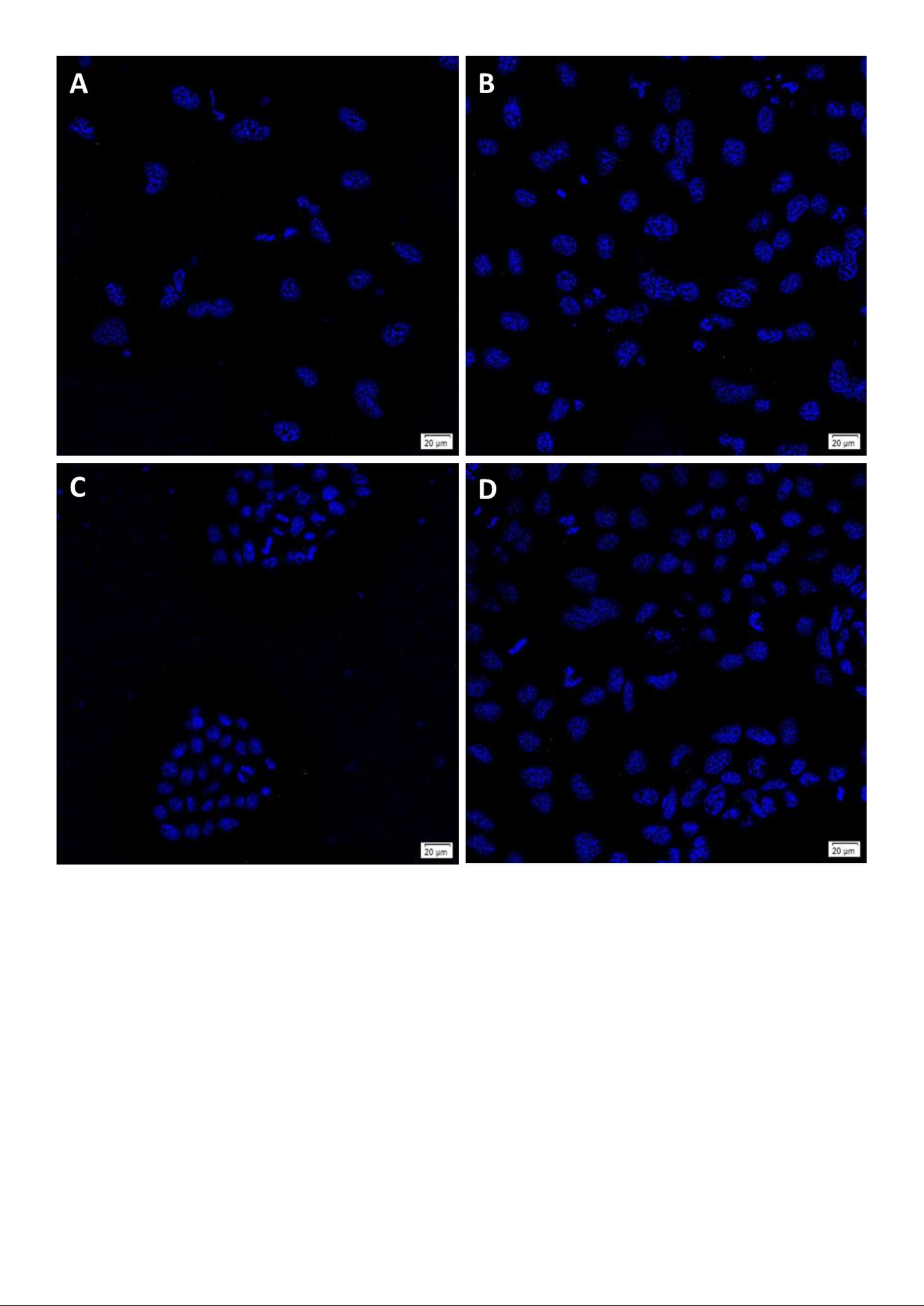

Supplement: S1 Fig — Negative controls were run for the control cells by omitting the primary antibodies for SSEA4/OCT4 (A) and for SOX2/TRA-1-60 (B) in NTERA-2 cells, and for SSEA4/OCT4 (C) and for SOX2/TRA-1-60 (D) in CaCo2 cells. Original magnification = 400x; scale bar = 20 μm. (TIF) [file pone.0232934.s001.tif]

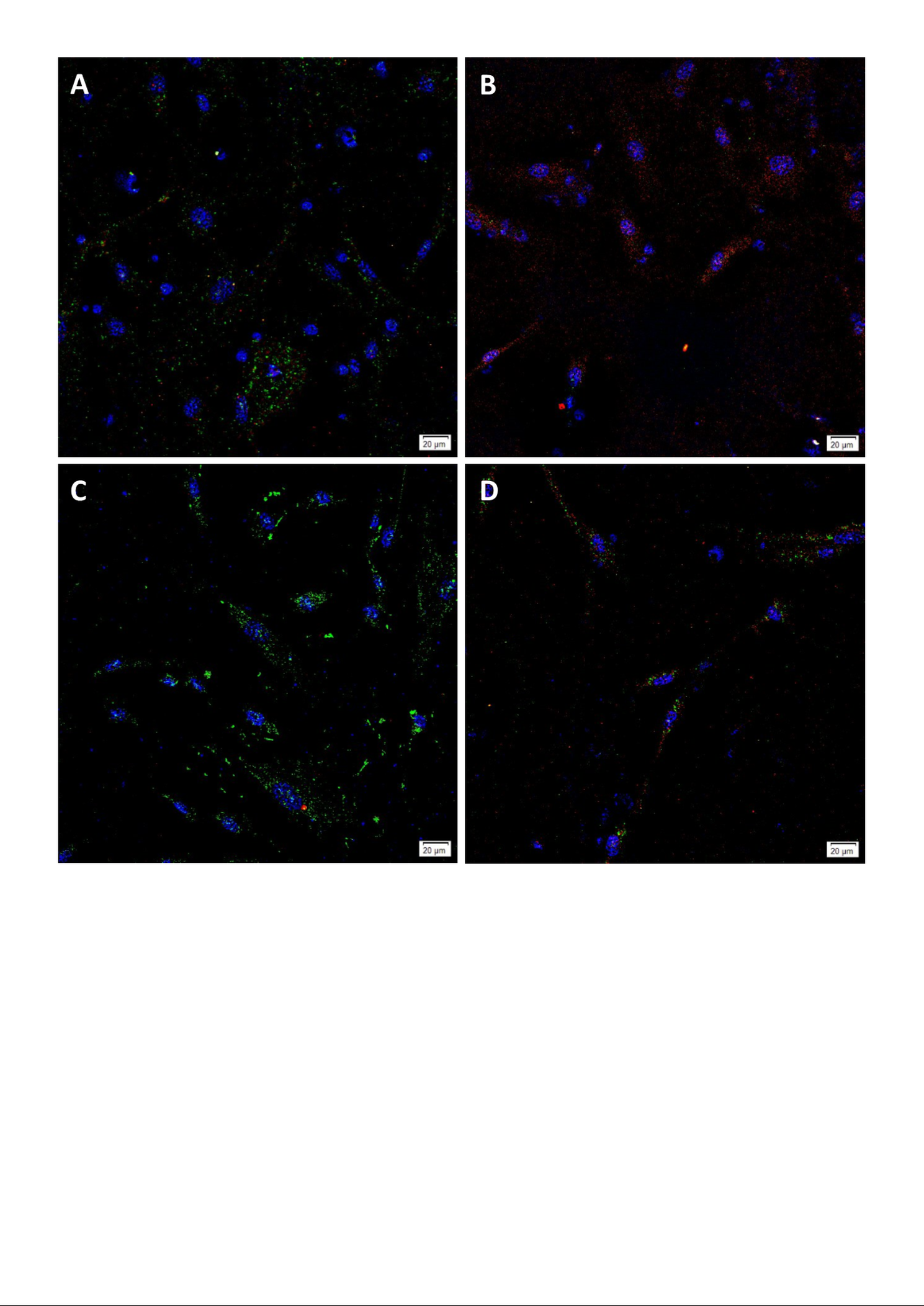

Supplement: S2 Fig — EpCAMLow cells from sample LG1 were stained for SSEA4 (green; A) and OCT4 (red; A), and for SOX2 (green; C) and TRA-1-60 (red; C). EpCAMHigh cells from sample LG1 were stained for SSEA4 (green; B) and OCT4 (red; B), and for SOX2 (green; D) and TRA-1-60 (red; D). Original magnification = 400x; scale bar = 20 μm. (TIF) [file pone.0232934.s002.tif]

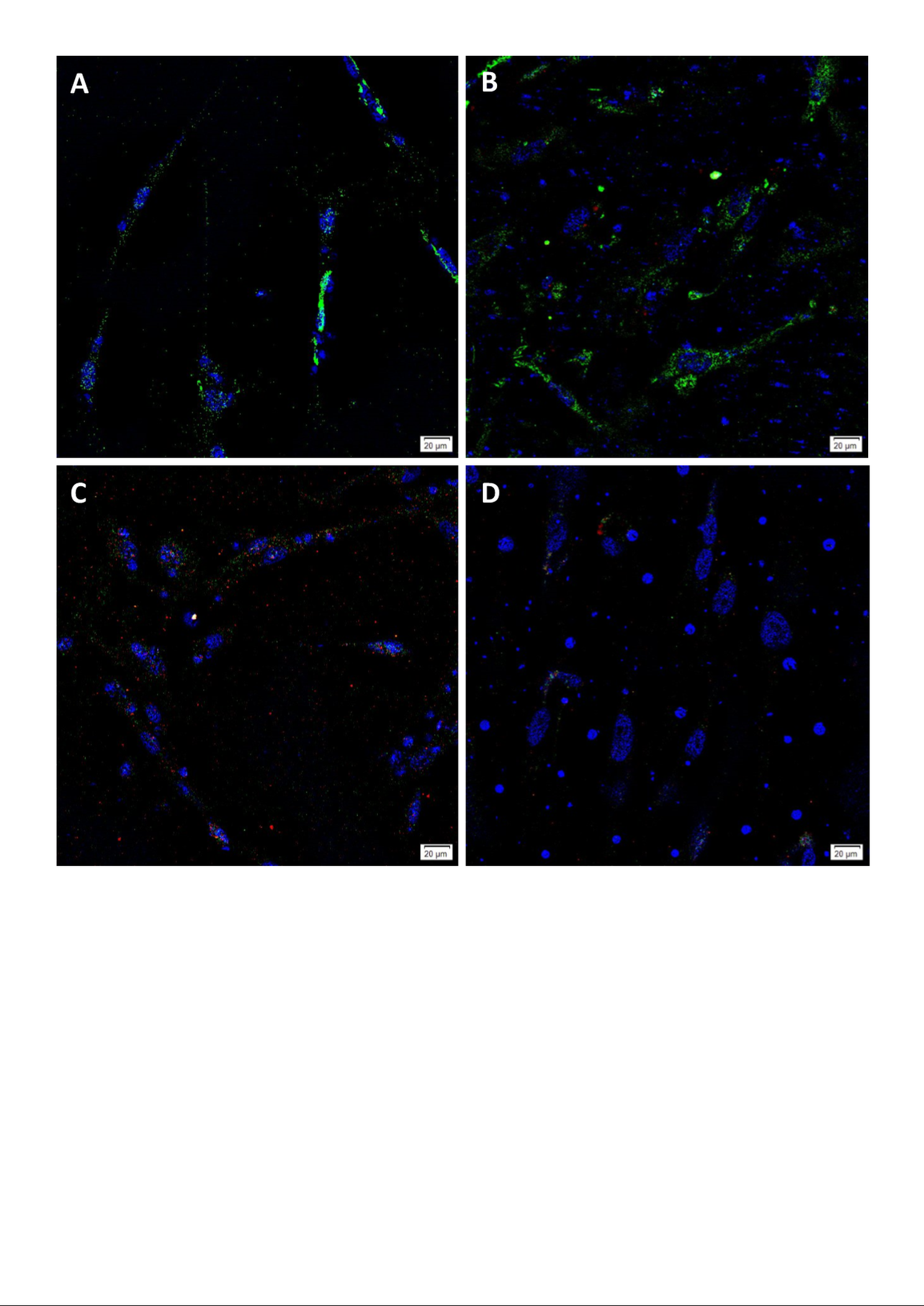

Supplement: S3 Fig — EpCAMLow cells from sample LG2 were stained for SSEA4 (green; A) and OCT4 (red; A), and for SOX2 (green; C) and TRA-1-60 (red; C). EpCAMHigh cells from sample LG2 were stained for SSEA4 (green; B) and OCT4 (red; B), and for SOX2 (green; D) and TRA-1-60 (red; D). Original magnification = 400x; scale bar = 20 μm. (TIF) [file pone.0232934.s003.tif]

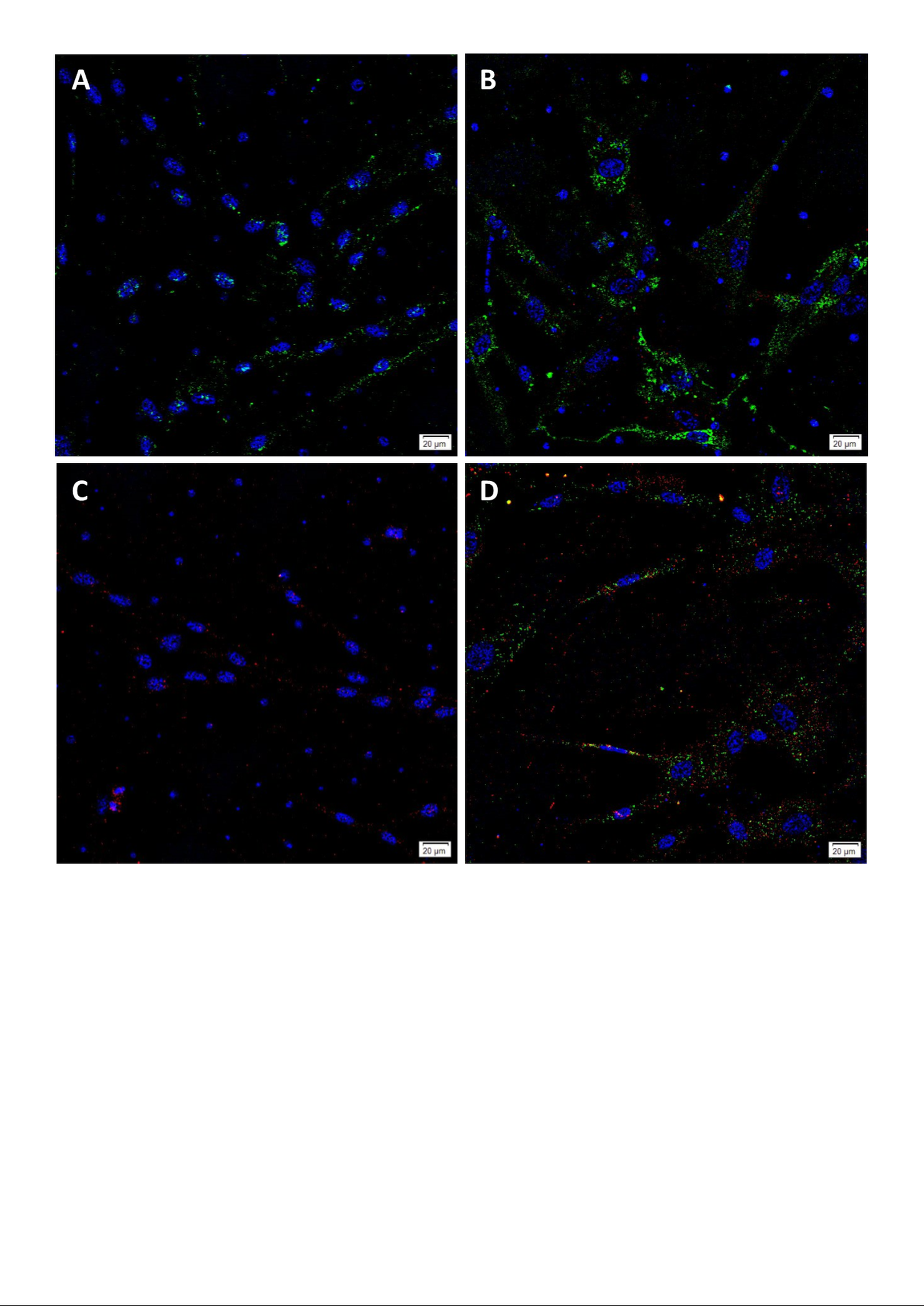

Supplement: S4 Fig — EpCAMLow cells from sample LG3 were stained for SSEA4 (green; A) and OCT4 (red; A), and for SOX2 (green; C) and TRA-1-60 (red; C). EpCAMHigh cells from sample LG3 were stained for SSEA4 (green; B) and OCT4 (red; B), and for SOX2 (green; D) and TRA-1-60 (red; D). Original magnification = 400x; scale bar = 20 μm. (TIF) [file pone.0232934.s004.tif]

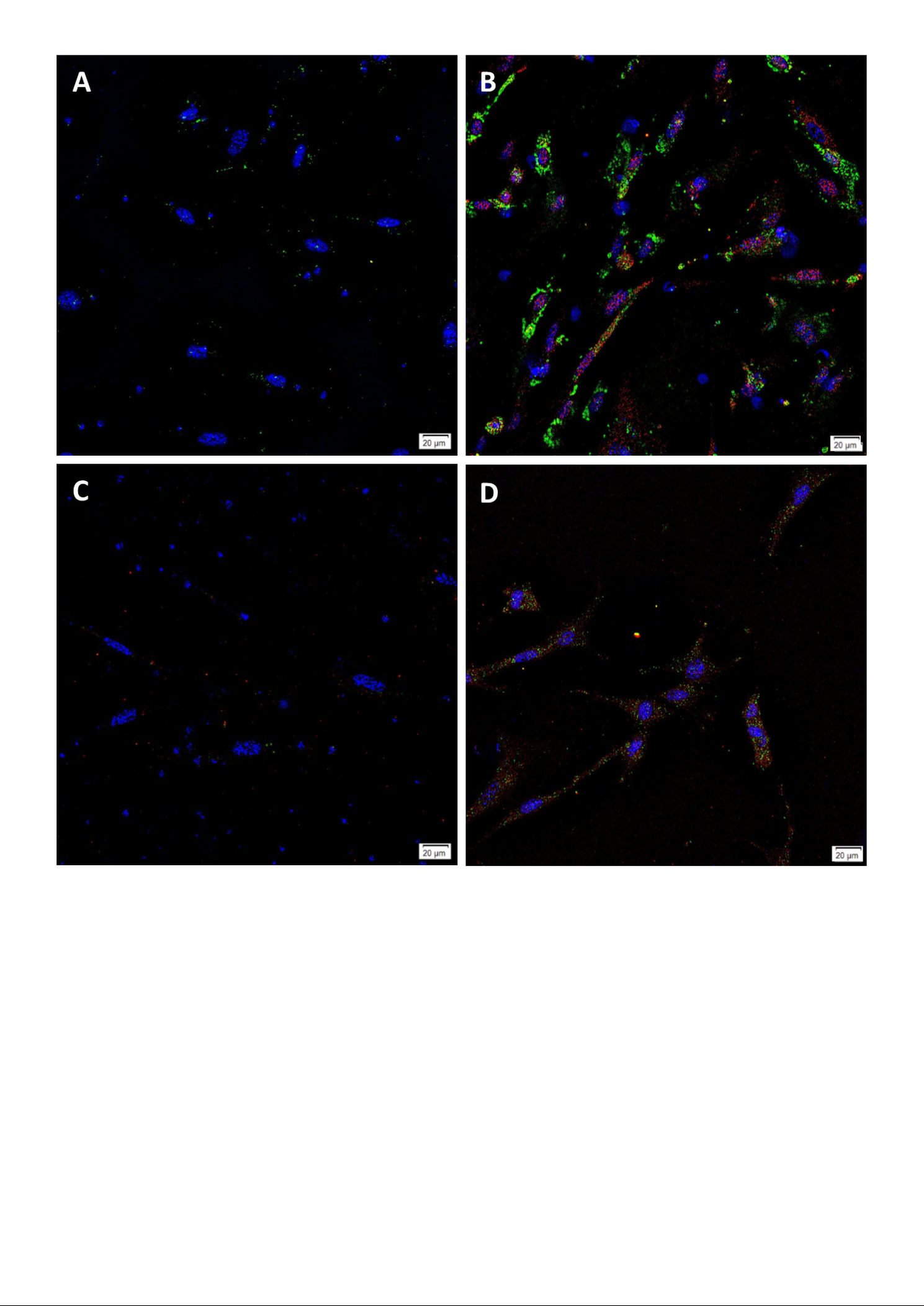

Supplement: S5 Fig — EpCAMLow cells from sample HG1 were stained for SSEA4 (green; A) and OCT4 (red; A), and for SOX2 (green; C) and TRA-1-60 (red; C). EpCAMHigh cells from sample HG1 were stained for SSEA4 (green; B) and OCT4 (red; B), and for SOX2 (green; D) and TRA-1-60 (red; D). Original magnification = 400x; scale bar = 20 μm. (TIF) [file pone.0232934.s005.tif]

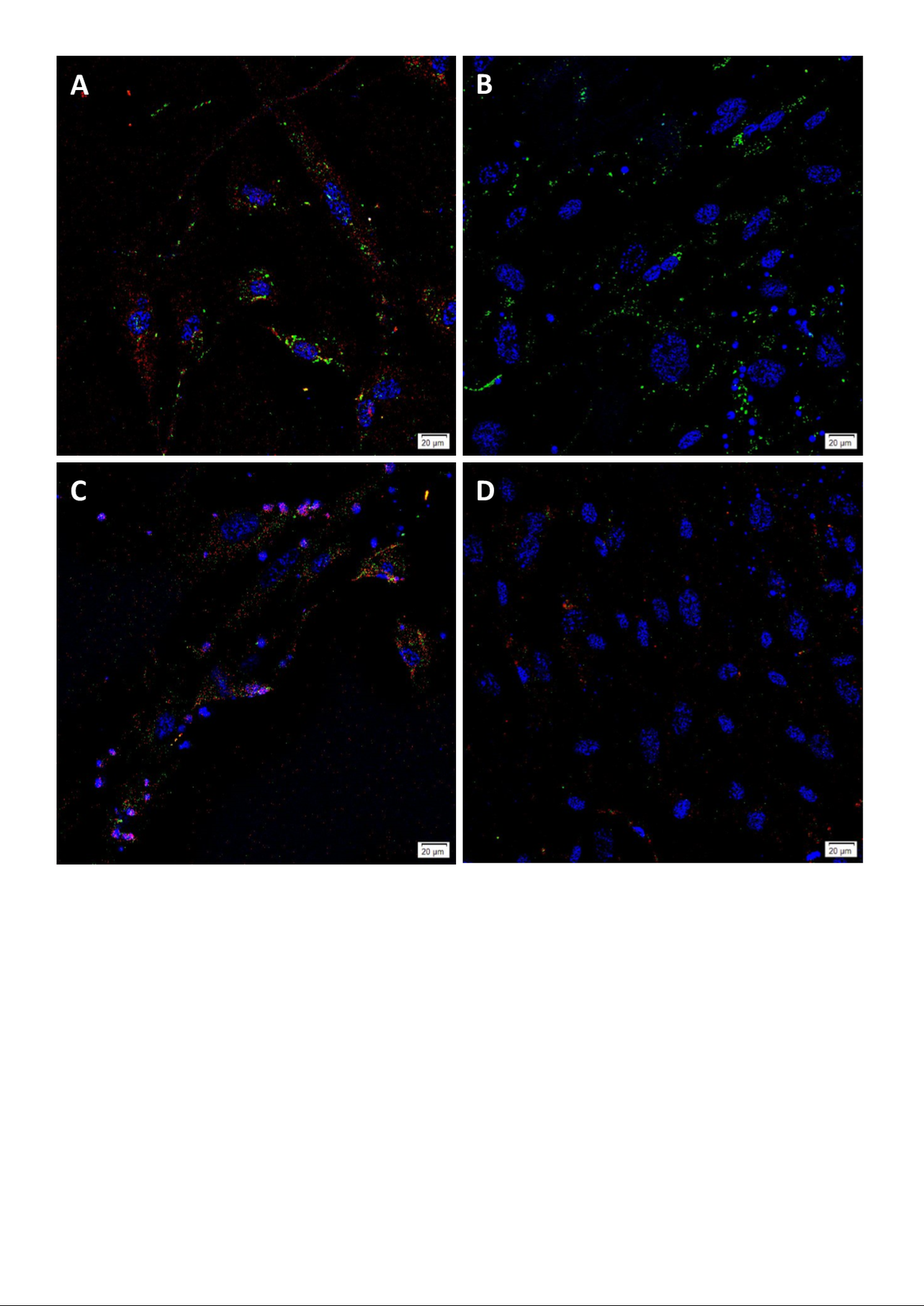

Supplement: S6 Fig — EpCAMLow cells from sample HG2 were stained for SSEA4 (green; A) and OCT4 (red; A), and for SOX2 (green; C) and TRA-1-60 (red; C). EpCAMHigh cells from sample HG2 were stained for SSEA4 (green; B) and OCT4 (red; B), and for SOX2 (green; D) and TRA-1-60 (red; D). Original magnification = 400x; scale bar = 20 μm. (TIF) [file pone.0232934.s006.tif]

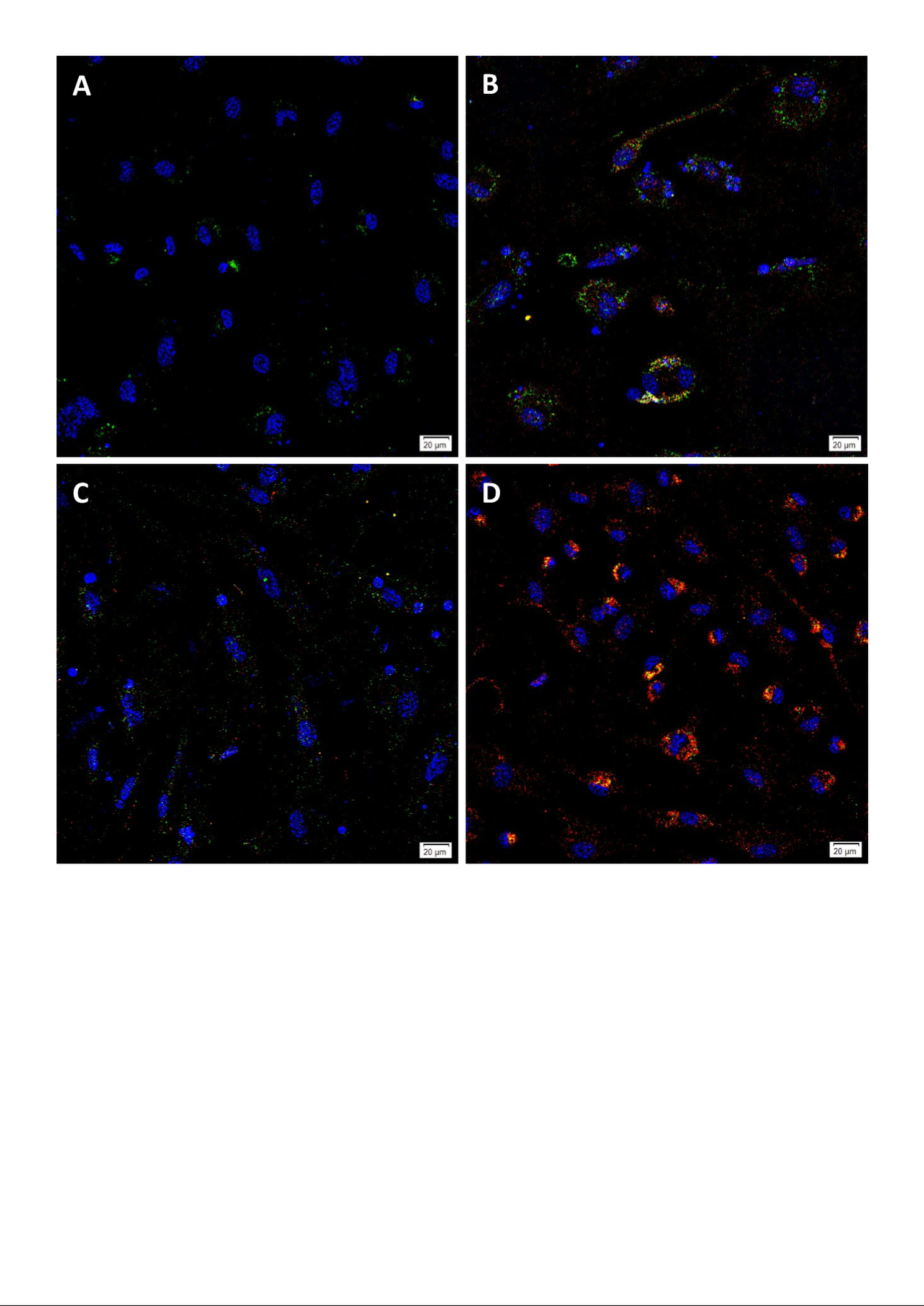

Supplement: S7 Fig — EpCAMLow cells from sample HG3 were stained for SSEA4 (green; A) and OCT4 (red; A), and for SOX2 (green; C) and TRA-1-60 (red; C). EpCAMHigh cells from sample HG3 were stained for SSEA4 (green; B) and OCT4 (red; B), and for SOX2 (green; D) and TRA-1-60 (red; D). Original magnification = 400x; scale bar = 20 μm. (TIF) [file pone.0232934.s007.tif]

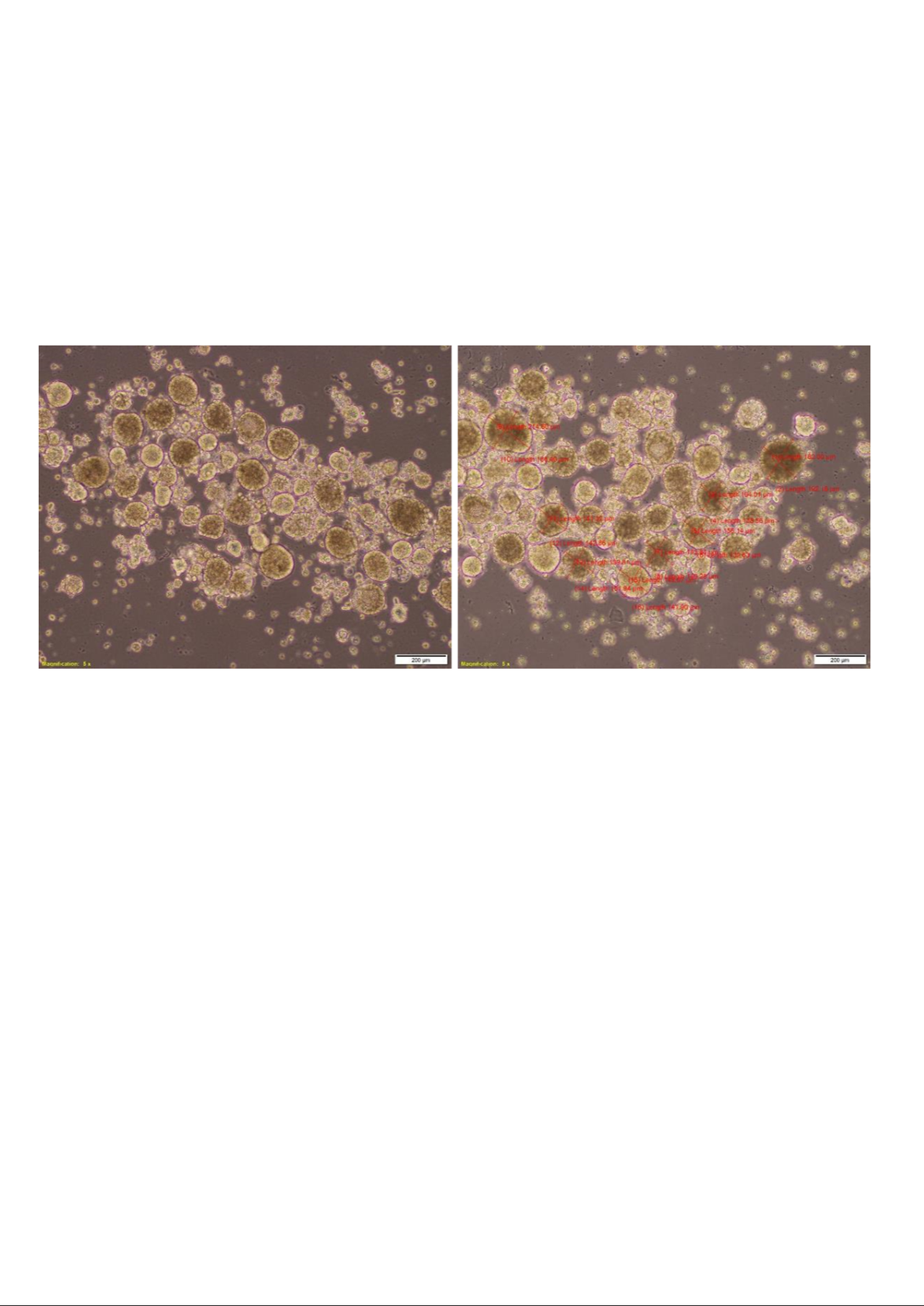

Supplement: S8 Fig — CaCo2 cells were used as a positive control for tumorsphere formation assays, here showing tumorsphere formation at day 7. Original magnification = 100x; scale bar = 200 μm. (TIF) [file pone.0232934.s008.tif]

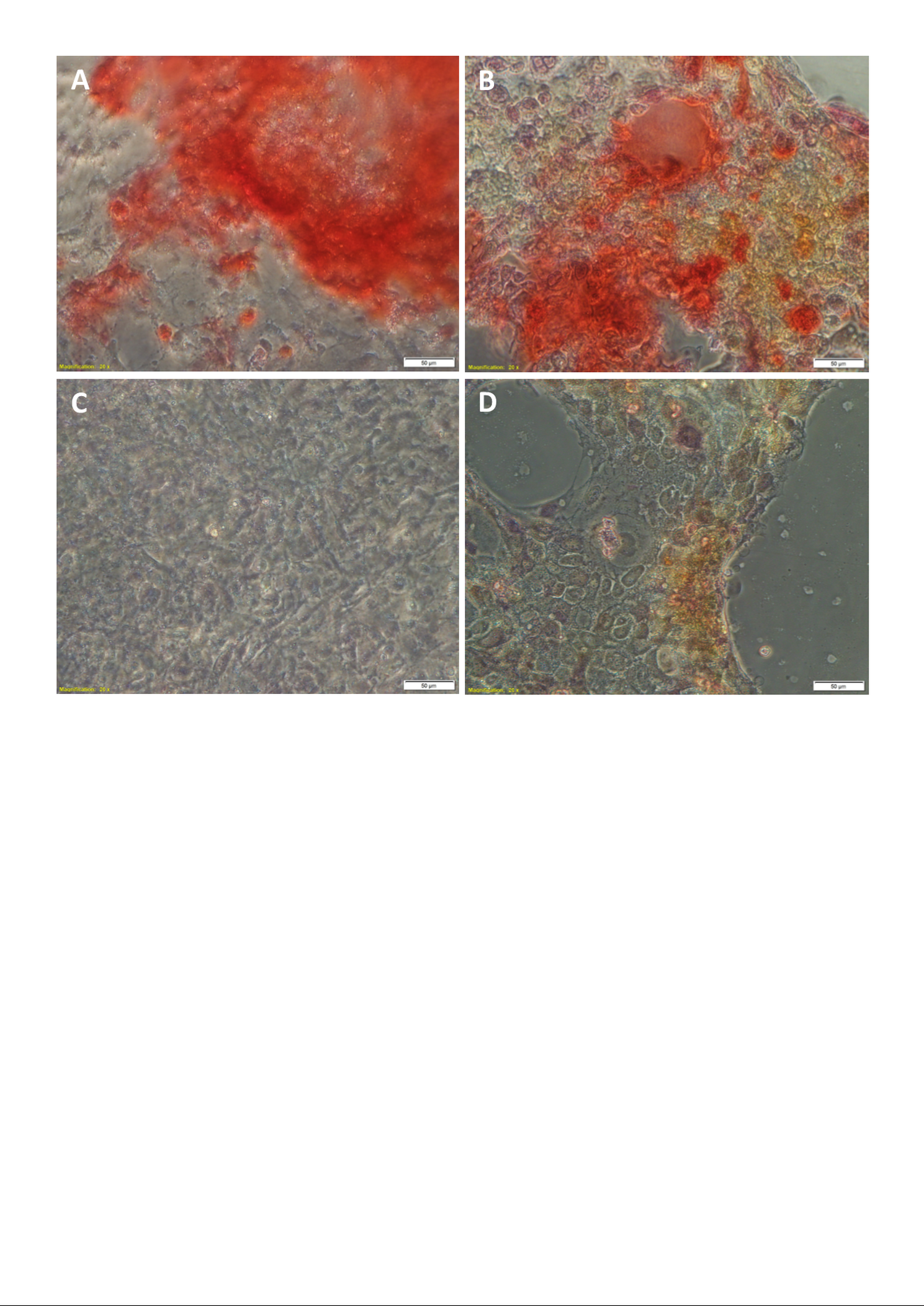

Supplement: S9 Fig — 3T3 cells (A) and CaCo2 cells (B) were used as positive controls for mesodermal differentiation. Negative controls were run by growing 3T3 cell (C) and CaCo2 cells (D) in regular culture media rather than differentiation media. Original magnification = 40x; scale bar = 50 μm. (TIF) [file pone.0232934.s009.tif]

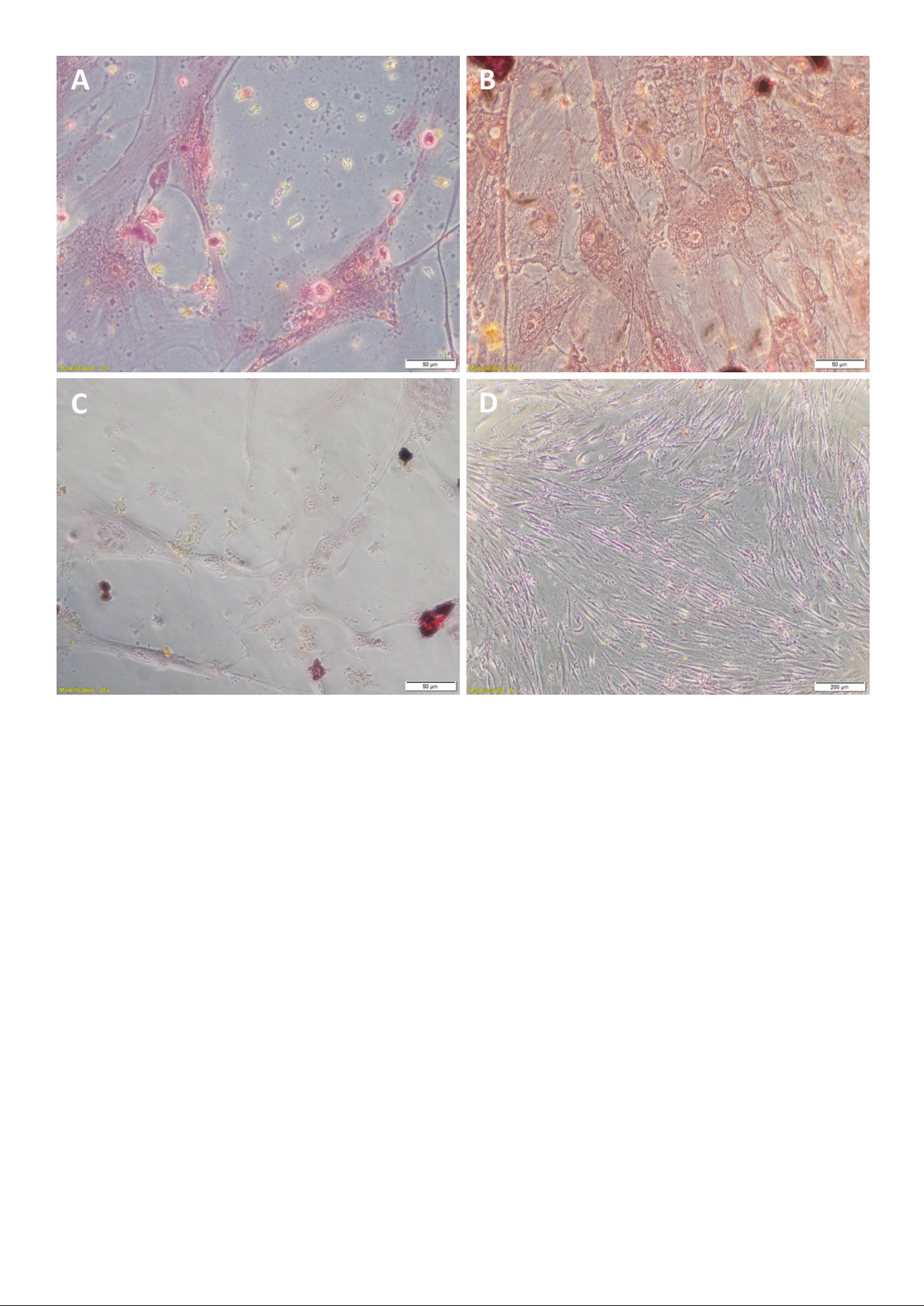

Supplement: S10 Fig — As a negative control, primary tumor-derived EpCAMLow (A) and EpCAMHigh (B) LGCA cells, and EpCAMLow (C) and EpCAMHigh (D) HGCA cells, were grown in regular culture media rather than differentiation media before being exposed to Alizarin Red (pH4.2). Original magnification = 40x; scale bar = 50 μm. (TIF) [file pone.0232934.s010.tif]

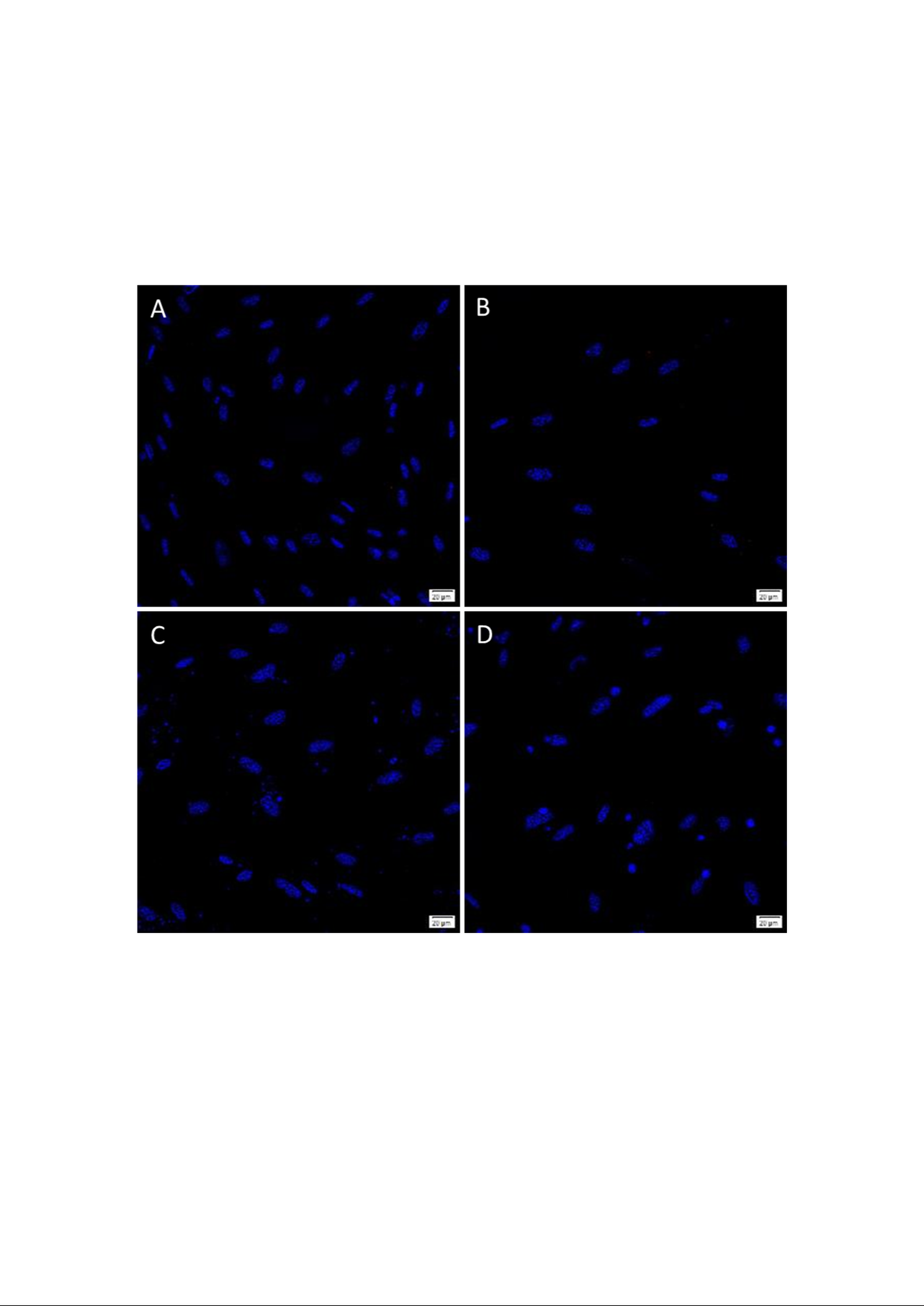

Supplement: S11 Fig — Negative controls were run for EpCAMLow (A) and EpCAMHigh (B) LGCA cells, and EpCAMLow (C) and EpCAMHigh (D) HGCA cells by omitting the anti-SOX17 primary antibody. Original magnification = 400x; scale bar = 20 μm. (TIF) [file pone.0232934.s011.tif]

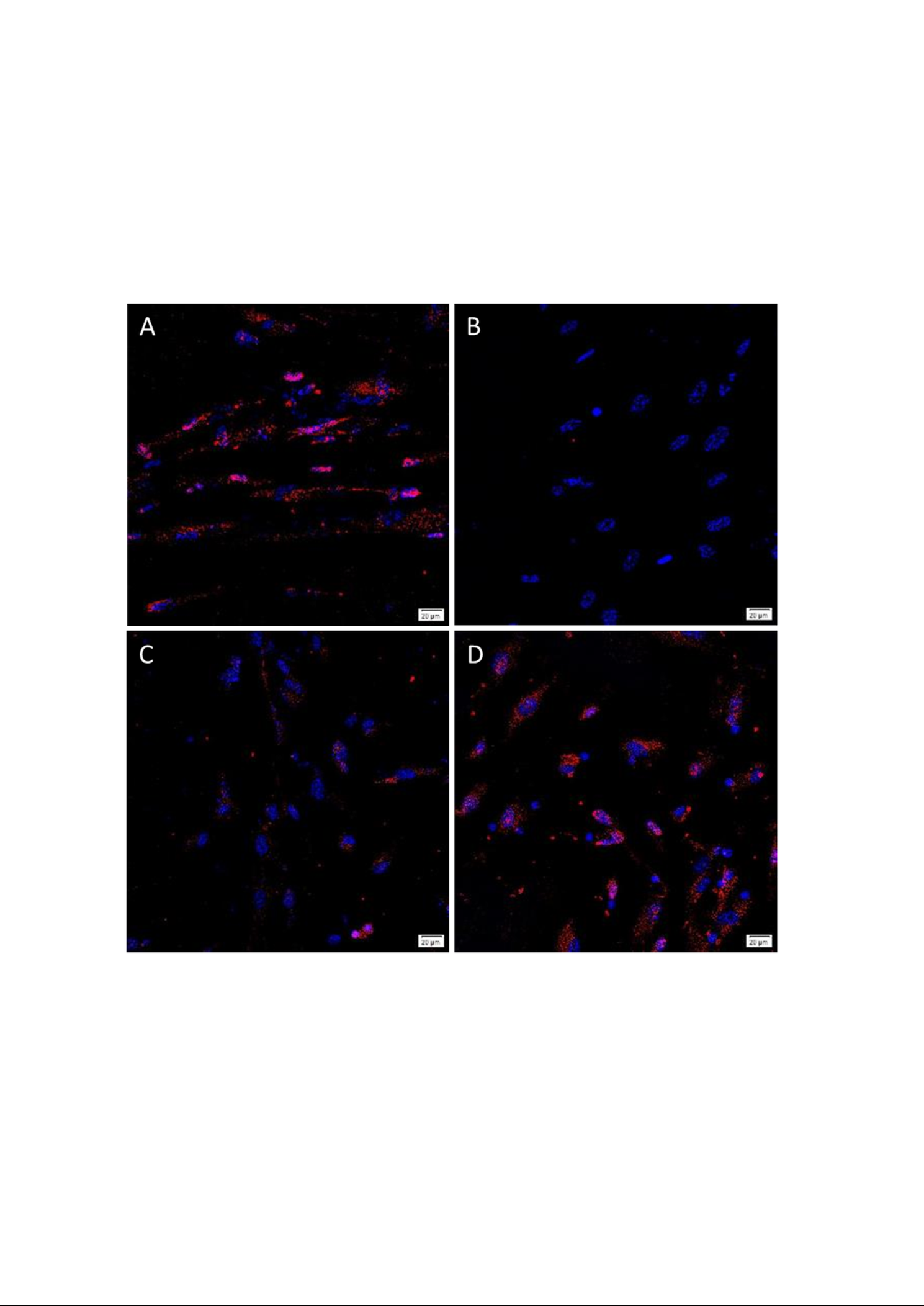

Supplement: S12 Fig — Negative controls were run for EpCAMLow (A) and EpCAMHigh (B) LGCA cells, and EpCAMLow (C) and EpCAMHigh (D) HGCA cells by growing them in regular culture media rather than differentiation media before being exposed to anti-SOX17. Original magnification = 400x; scale bar = 20 μm. (TIF) [file pone.0232934.s012.tif]

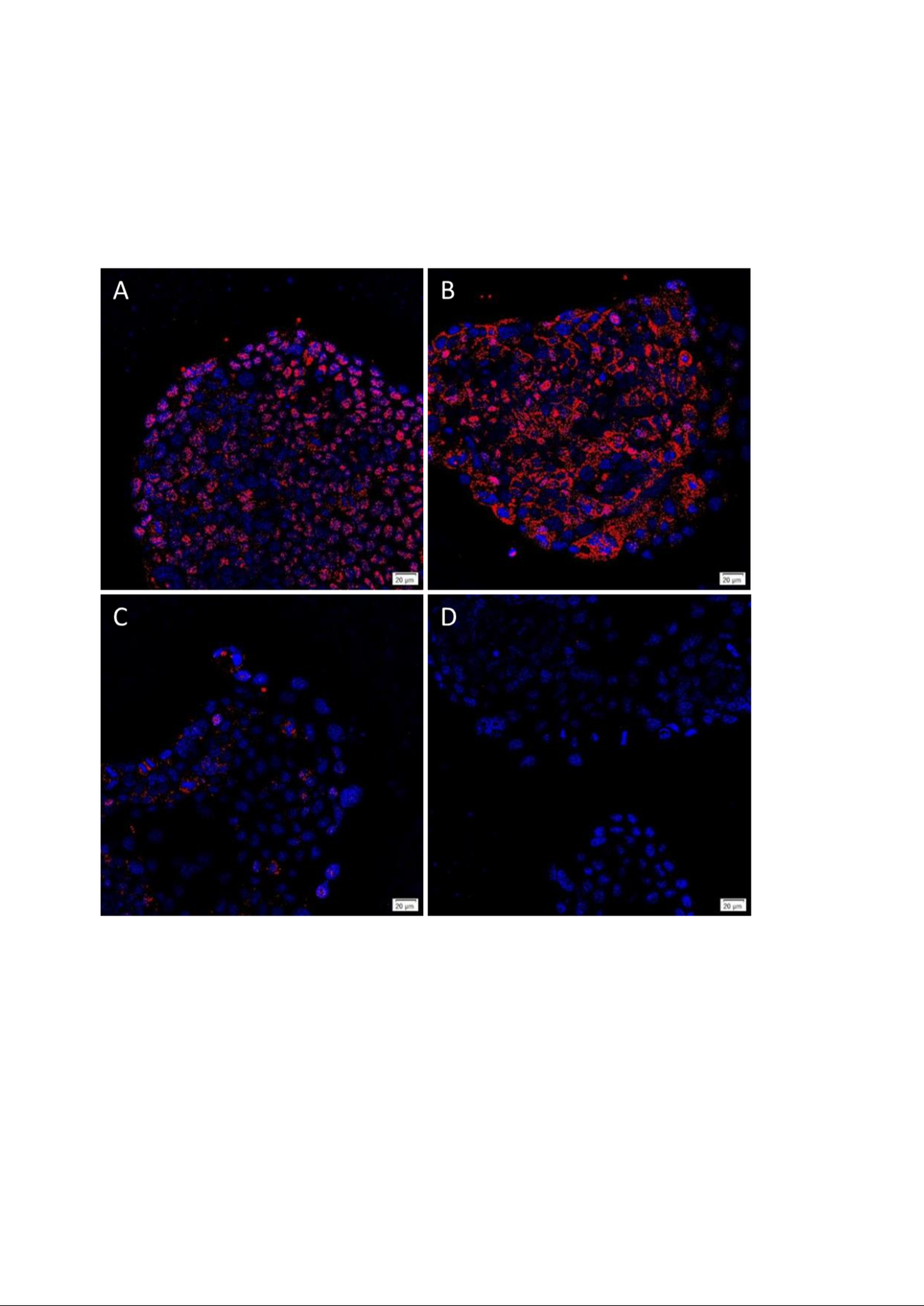

Supplement: S13 Fig — CaCo2 cells were used as a positive control for endoderm differentiation (A). CaCo2 were also grown in regular culture media as a control (B and C). A negative control was run by omitting the anti-SOX17 primary antibody from CaCo2 cells grown in differentiation media (D). Original magnification = 400x; scale bar = 20 μm. (TIF) [file pone.0232934.s013.tif]

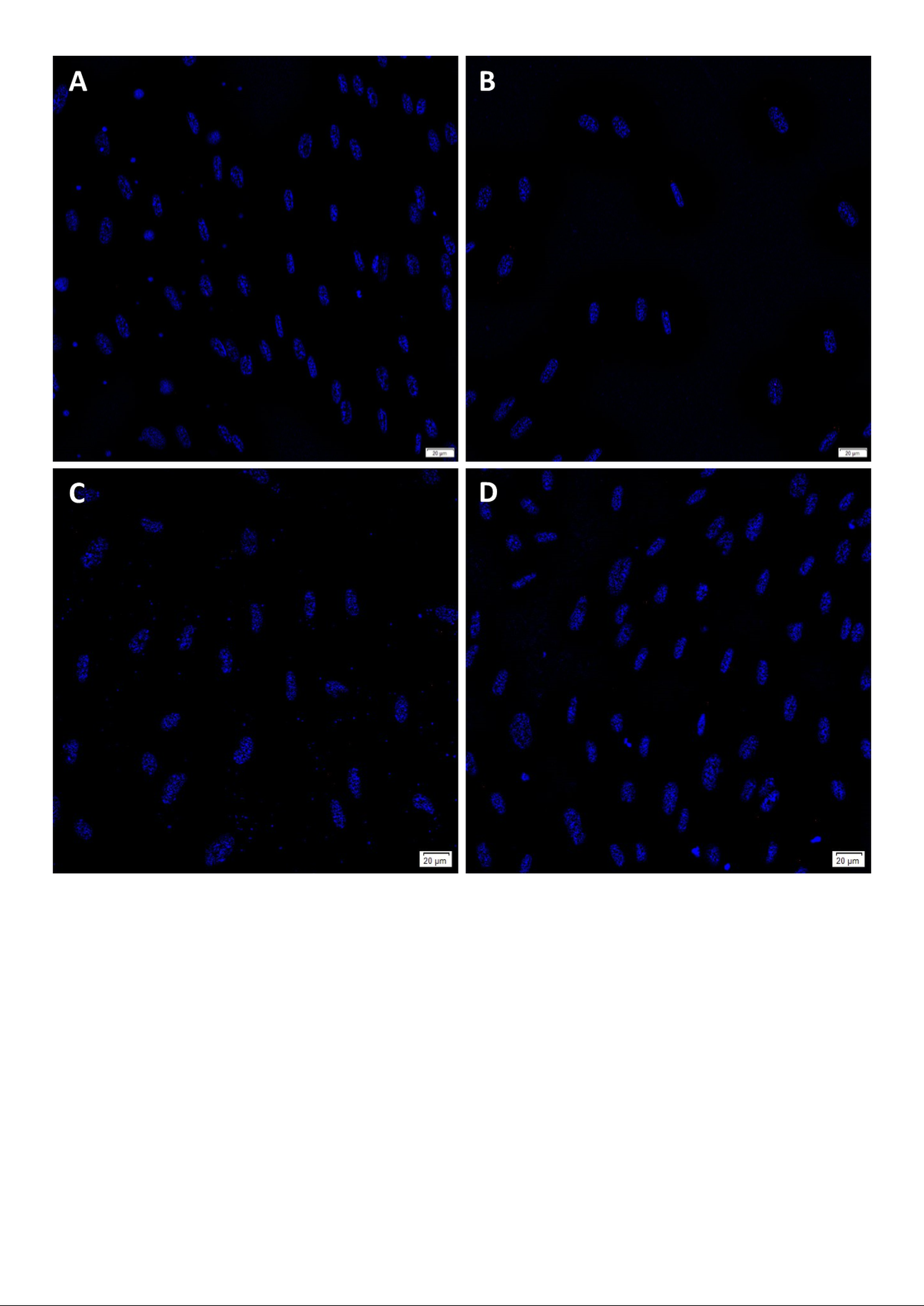

Supplement: S14 Fig — Negative controls were run for EpCAMLow (A) and EpCAMHigh (B) LGCA cells, and EpCAMLow (C) and EpCAMHigh (D) HGCA cells by omitting the anti-Otx2 primary antibody. Original magnification = 400x; scale bar = 20 μm. (TIF) [file pone.0232934.s014.tif]

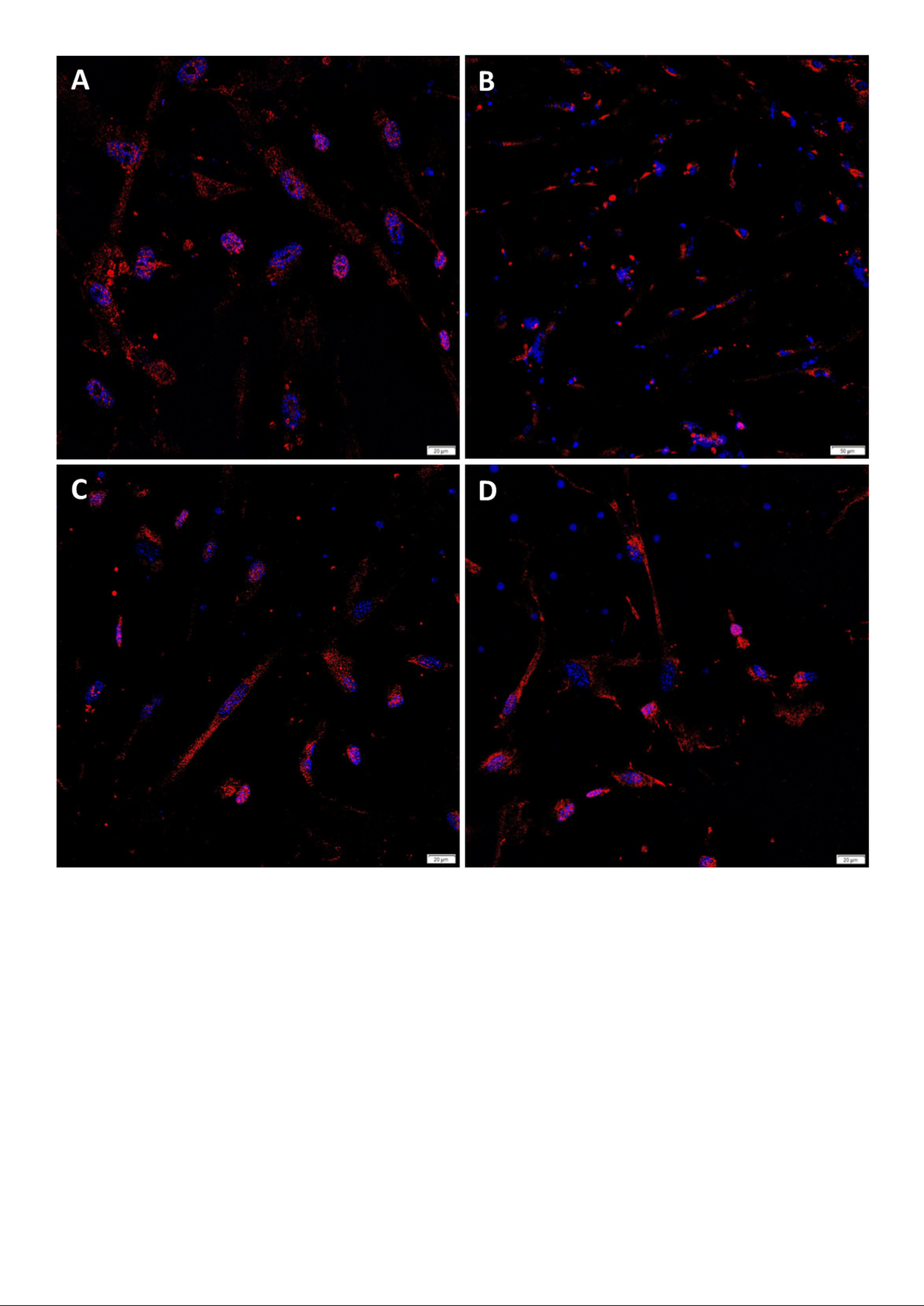

Supplement: S15 Fig — Negative controls were run for EpCAMLow (A) and EpCAMHigh (B) LGCA cells, and EpCAMLow (C) and EpCAMHigh (D) HGCA cells by growing them in regular culture media rather than differentiation media before being exposed to anti-Otx2. Original magnification = 400x; scale bar = 20 μm. (TIF) [file pone.0232934.s015.tif]

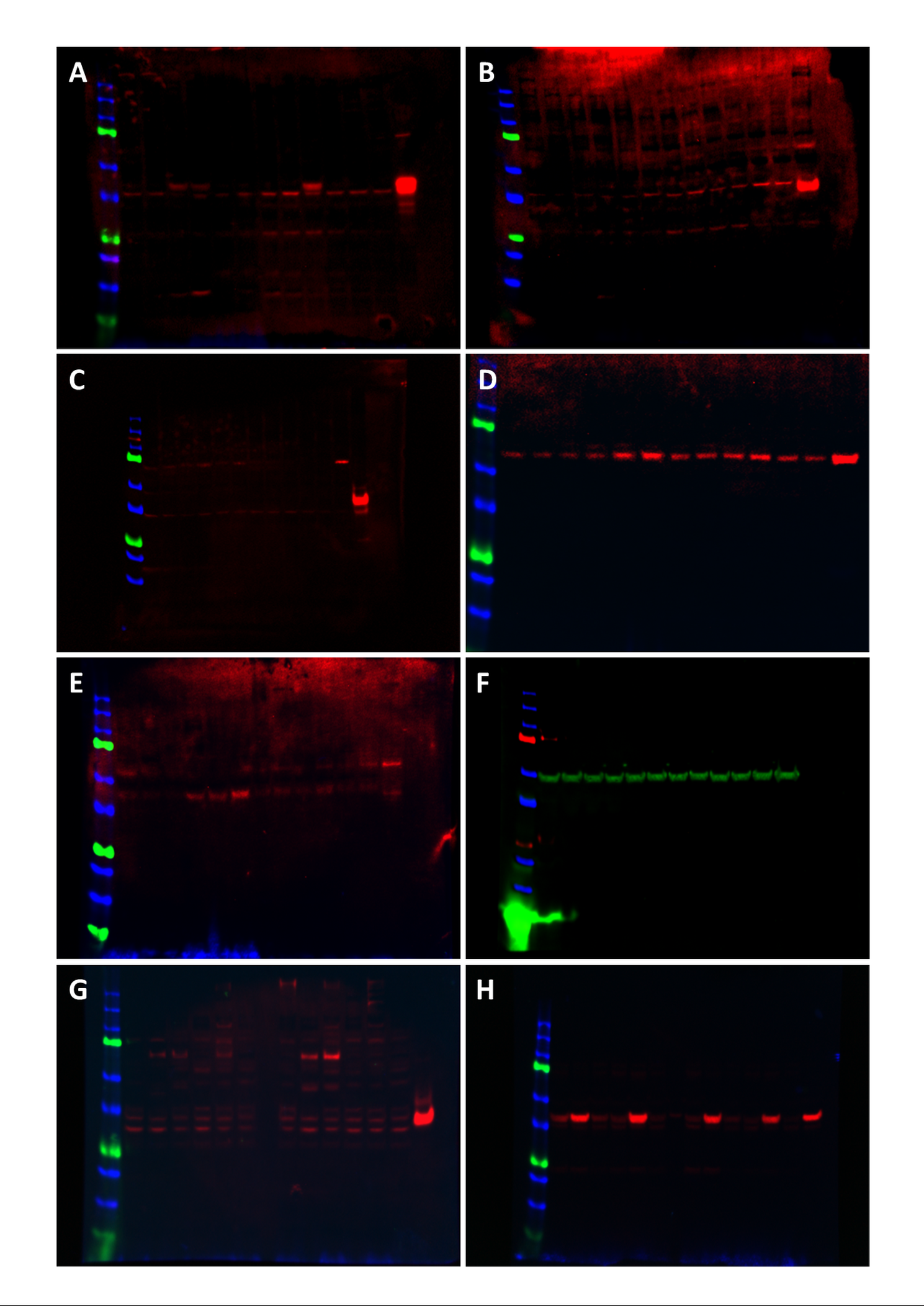

Supplement: S17 Fig — Uncropped images of western blotting membranes before adjusting for background fluorescence, showing OCT4 (A), SOX2 (B), NANOG (C), KLF4 (D), c-MYC (E), α-tubulin (F), EpCAM (G) and α-SMA (H). (TIF) [file pone.0232934.s017.tif]
